# A Tale of Two States: Similarities and Differences in Neuropeptide Signaling in Larval and Adult *Drosophila*

**DOI:** 10.1002/cne.70218

**Published:** 2026-10-22

**Authors:** Dick R. Nässel

**Affiliations:** ^1^ Department of Zoology Stockholm University Stockholm Sweden

## Abstract

Having the life cycle divided into a larval and an adult stage, each dwelling in a separate habitat, is advantageous since it prevents competition for resources and separates development and reproduction into different stages. In holometabolous insects, larval life is characterized by intense feeding, growth, and molting, whereas the adults constitute the reproductive and highly mobile stage. This review discusses how this switch in lifestyle is reflected in the neuroendocrine system and peptidergic signaling. Neuropeptides and peptide hormones (collectively NPHs) are important regulators of most aspects of development and daily life physiology and behavior. Thus, it is of interest to understand to what extent peptidergic neurons and NPHs signaling change as the behavior and physiology alters and diversifies after metamorphosis. Focusing on larvae and adults of *Drosophila melanogaster*, I discuss changes in signaling in peptidergic systems. As an example, the complement of neurosecretory cells and their NPHs remain largely the same over metamorphosis, yet their functional roles change to a large extent. In larvae, many NPHs are mainly associated with regulation of development and growth, whereas in adults they regulate reproduction and complex behavior. Common to the two stages, NPHs regulate feeding, metabolism, excretion, and avoidance behavior. Other peptidergic systems, such as components of the biological clock increase drastically in complexity, both in terms of neuron types and NPHs. In summary, NPH signaling is highly flexible and individual NPHs serve multiple functions, many of which are stage specific. This review provides comprehensive descriptions of structure and function of major peptidergic systems in larvae and adults.

## Graphical abstract

Neurosecretory cells of the brain are mostly the same in larvae and adults of *Drosophila melanogaster* and they produce the same neuropeptides and peptide hormones (NPHs), yet their functional roles differ to a large extent between the two stages. Many other peptidergic neurons change both in numbers and function.

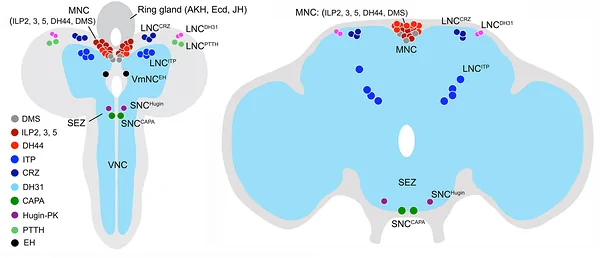

**Abbreviations**
AKH: adipokinetic hormone
Ast‐A: allatostatin‐A
Ast‐C: allatostatin‐C
Burs: bursicon (Burs)
CAPA
: capability (CAPA‐PVK1 and PVK2, Capa‐pyrokinin)
CCAP: crustacean cardioactive peptide
CCHa: CCHamide‐1 and 2 (CCHa‐1 and 2)
CNMa: CNMamide
CRZ: corazonin
DH31: diuretic hormone 31
DH44: diuretic hormone 44
DMS: dromyosuppressin
DSK: drosulfakinin
EH: eclosion hormone
ETH: ecdysis triggering hormone
FMRFa: FMRFamide
GPB5: glycoprotein B5
Hugin‐PK: Hugin‐pyrokinin (Hug‐PK)
ILP: insulin‐like peptide (ILP1‐8)
ITP: ion transport peptide
LK: leucokinin
MIP: myoinhibitory peptide (Allatostatin‐B)
NPF: neuropeptide F
NPLP1: neuropeptide‐like precursor 1
NTL: natalisin
OK: orcokinin (OK‐A and B)
PBAN: pigment biosynthesis activating neuropeptide
PDF: pigment‐dispersing factor
Proct: proctolin
PTTH: prothoracicotropic hormone
RYa: RYamide
SIFa: SIFamide
sNPF: short NPF
SP: sex peptide
TK: tachykinin

## 1 Introduction

*There is only one problem in the world, and that is:… How does one burst the cocoon and become a butterfly?*

Thomas Mann, *Doctor Faustus* (1947)

In many animal taxa, the life cycle is divided into a larval and an adult stage with drastically different morphologies and lifestyles. This separation enables dwelling in different habitats and thereby prevents competition for resources, separates development and reproduction into different life stages, allows for more rapid growth, and may also reduce the risk of predation (Brown and Cai 2007; Manthey et al. 2024; Rolff et al. 2019; Rose 2005; Truman 2019; Truman and Riddiford 2019). Among the most well‐studied organisms that display metamorphosis are the holometabolous insects (Rolff et al. 2019; Suzuki 2026; Truman 2019; Truman and Riddiford 2019). In these insects, the larval stages are worm‐like organisms that differ extensively both morphologically and behaviorally from their more advanced adult forms, the winged imagos. The two developmental stages are separated by an immobile and non‐feeding pupal stage, together constituting a tripartite development. The larva is a feeding and growth stage, the pupa is where most differentiation of adult structures occurs (metamorphosis), and the imago is the highly mobile reproductive stage. Furthermore, larval development is commonly divided up into several stages, termed instars, each triggered by a shedding of the old cuticle (molting) to enable growth. Larval growth, and each molt, is controlled by hormones, and molting engages specific motor programs to shed the old cuticle (Callier and Nijhout 2013; Ewer 2005; Truman and Riddiford 2019; Zitnan and Adams 2005).

While many adult tissues are newly generated from nests of stem cells (imaginal discs) during metamorphosis, a considerable number of neurons persist from the larval to the adult stage, but they undergo extensive remodeling during metamorphosis (Tissot and Stocker 2000; Truman and Williams 2026). At the same time, a large number of adult neurons are generated from neuroblasts during the pupal stage. The larval brain and ventral nerve cord (VNC) that constitute the central nervous system (CNS) support a much simpler physiology and behavior than that of the adult. It is mainly concerned with controlling activity, simple locomotion (including avoidance), feeding, metabolism, growth, and development. The larval neuroendocrine system of the brain and retrocerebral complex (ring gland) primarily regulates nutrient‐dependent growth and metabolism, but also in alliance with the clock, times development and molts (Ewer 2005; Hückesfeld et al. 2021; Truman and Riddiford 2019). In comparison, the adult brain is much larger and far more complex (more modular), matching the increased complexity and new aspects of behavior and physiology. The most striking examples are the metamorphic *de novo* development of the optic lobes that serve the compound eyes and advanced visual information processing (Nern et al. 2025) and the extensive elaboration of the biological clock network (Reinhard et al. 2024). Another feature that characterizes the adult brain in *Drosophila* is sexually dimorphic circuits that control social behavior (e.g., mating and aggression; Auer and Benton 2016; Berg et al. 2026; Dickson 2008; Kim and Pan 2026; Pavlou and Goodwin 2013); also in the VNC sex‐specific circuitry develops (Matsliah et al. 2026). Morphologically, the neuroendocrine systems in the brain and VNC change less during metamorphosis, yet obtain multiple novel functions. Whereas the number of brain neurons increases drastically, the complement of neuropeptides and peptide hormones (collectively NPHs) is largely the same in larvae and adults. However, as I will show in this review, the functions of most studied NPHs multiply and change drastically during metamorphosis.

This review summarizes how the peptidergic systems change structurally and functionally over metamorphosis and describes the mostly distinct functions of NPHs in larvae and adults. It is important to note that in most cases, each of the NPHs is found in multiple cell types. Thus, a given NPH can be produced by NSCs, sensory cells, interneurons, efferent neurons (including motoneurons), and gut endocrine cells (enteroendocrine cells, EECs), as well as by other cell types (see Nässel and Zandawala 2019). In most cases, it is not clear whether the functions or actions of a specific NPH produced in diverse cell types are coordinated (orchestrating) or if their functions are uncoupled (distributed) temporarily, spatially and/or qualitatively. Actually, it is likely that many neuropeptides produced by interneurons act in different ways depending on the circuit. Thus, for instance, tachykinins (TKs) and short neuropeptide F (sNPF) are produced in numerous neurons of many different types and may act in specific ways depending on circuit and colocalized small‐molecule neurotransmitters (SMNs) and neuropeptides (see Nässel 2018; Nässel, Zandawala, et al. 2019). A recent paper clearly demonstrated this circuit‐specific functional specificity for neuropeptide F (NPF) in *Drosophila* (Endres et al. 2026), where the peptide can convey both excitatory and inhibitory responses depending on circuit position and target. This multiplicity of possible actions means that assigning functional roles to many of the individual NPHs is a difficult task and makes comparisons between larval and adult functions more complex (many interneuronal neuropeptides do not display *single* actions or functions). Note that I occasionally describe roles of NPH as *actions* when they act on specific targets and as *functions* in cases where coordinated actions lead to a behavioral or physiological outcome, but in the tables, I occasionally use the more generic term *roles* to simplify the text. Focus in this review is on developmental changes in peptidergic signaling in *Drosophila melanogaster* since most data are available for this species, but the relevance of the principles discussed are likely to be common to most organisms that undergo complete metamorphosis. One important caveat is that there is an imbalance in the number of studies dealing with NPH roles in physiology and behavior in *Drosophila* larvae, compared to the extensively investigated adults. Hopefully, this review will trigger an interest in extending our understanding of larval NPH function and maybe uncover further differences and similarities in NPH functions in the two stages.

In the following, I first summarize some general aspects of larval and adult lifestyles and general aspects of NPH signaling. Next, I describe neurosecretory cells (NSCs) systems and their functional roles in the two stages, first those of the brain and then the VNC. The ensuing sections deal with NPHs involved in ecdysis and thereafter compare NPH function in the clock network, intestinal EECs, and finally in foraging and feeding in larvae and adults. Note that I have not specifically addressed NPH receptors in the two stages (e.g., distribution and functional subtypes), but where relevant functional contributions of receptors to NPH signaling have been studied, this is discussed.

## 2 Some General Aspects of Larval and Adult Lifestyle

### 2.1 Larva

Life of the larval insect differs prominently from that of the reproductive and highly mobile adult. The larva spends most of its time foraging and feeding, which involves also simple sensory processing and locomotion. The *Drosophila* larva has no bona fide eyes, only light‐sensitive stemmata (Bolwig organs) and a general photosensitivity supported by multidendritic neurons in the integument (Imambocus et al. 2022; Sprecher et al. 2011; Yamanaka, Romero, et al. 2013). The Bolwig organ photoreceptors are utilized in entrainment of the simple larval clock, as well as in phototaxis behavior, simple vision, and even visual learning (see e.g., Gerber et al. 2004; Justice et al. 2012; Larderet et al. 2017; Mazzoni et al. 2005; Poe et al. 2023; Yamanaka, Romero, et al. 2013). Furthermore, larvae are reacting to noxious stimuli (nociception), display escape behaviors, and react to infections and parasites (innate immunity). Most of these defensive processes involve NPHs (see An et al. 2012; Gu et al. 2022; Guntur et al. 2015; Y. Guo et al. 2025; Imambocus et al. 2025; Imambocus et al. 2022; Oikawa et al. 2023). The most extensively studied roles of NPHs are in regulation of growth and developmental timing (references in Tables 1 and 2). One intensively studied phenomenon is the peptidergic regulation of molting (ecdysis) and eclosion, which is coupled to the growth in insects (reviewed in Ewer 2005; Truman and Riddiford 2019; B. H. White and Ewer 2014). I do, however, not discuss this topic in great detail (or roles of NPHs in embryo and pupae) but focus mainly on the larval and the adult stages. The known roles of NPHs in larval behavior and physiology in *Drosophila* are listed in Table 2.

**TABLE 1:** Roles of NPHs specific to either larvae or adults in *Drosophila*.

Role/action	NPH	Specifics^a^	References
**Larva**			
Molting/ecdysis	AstA	Timing, ecdysis	(Deveci et al. 2019)
	AstC	Metamorphosis	(C. Li et al. 2024)
	Bursicon	Ecdysis cascade	(An et al. 2012; Diao et al. 2017)
	CCAP	Ecdysis cascade	(J. H. Park et al. 2003)
	EH	Ecdysis cascade	(McNabb et al. 1997; Scott et al. 2020)
	ETH	Ecdysis cascade	(Y. ‐J. Kim et al. 2006; B. H. White and Ewer 2014; Žitňan et al. 2007; Zitnan et al. 1996)
	FMRFa	Ecdysis cascade	(Y. ‐J. Kim et al. 2006)
	Hugin‐PK	Ecdysis cascade	(Meng et al. 2002)
	LK	Pre‐ecdysis, tracheal clearing	(D. H. Kim et al. 2015, 2018; Y.‐J. Kim et al. 2006)
	MIP	Glue expulsion	(Fernandez‐Acosta et al. 2024)
	PTTH	Adult eclosion	(McBrayer et al. 2007; Yamanaka, Rewitz, et al. 2013)
Other developmental processes (including growth regulation)	Bursicon	Growth, metabolism via DILPs	(Kubrak et al. 2024)
	CCHa2	Growth, metabolism via DILPs	(Sano et al. 2015)
	CRZ	Via PTTH and ecdysone	(Imura et al. 2020; Megha et al. 2019)
	ILP2, 3, 5	Growth, metabolism	(Brogiolo et al. 2001; Dunham and Bland 2026; Géminard et al. 2006; Grönke et al. 2010; Koyama and Mirth 2018; Okamoto and Mizoguchi 2026; Tennessen and Thummel 2011)
	ILP6	Growth	(Okamoto et al. 2009; Slaidina et al. 2009)
	ILP8	Growth, developmental timing	(Colombani et al. 2015; Colombani et al. 2012; Garelli et al. 2012; Garelli et al. 2015)
	GPA2/GPB5	Larval to pupal transition	(Vandersmissen et al. 2014)
	sNPF	Growth via ILPs (IPCs)	(K. S. Lee et al. 2009)
	TK	Growth via ILPs (IPCs)	(Meschi et al. 2019)
Regulation of hormone release (in control of growth and development)	AKH	DILP release (IPCs)	(S. K. Kim and Rulifson 2004)
	CCHa2	DILP2, 5 release (IPCs)	(Sano et al. 2015)
	CRZ	PTTH and Ecdysone	(Imura et al. 2020)
	Hugin‐PK	JH, via corpora allata	(Mizuno et al. 2021)
	ILP2,3,5	JH and Ecdysone	(Koyama and Mirth 2018; Moeller et al. 2017; Tu et al. 2005)
	ILP7	ILP release (IPCs)	(Prince et al. 2021)
	NPF	Ecdysone	(Kannangara et al. 2020)
	PTTH	Ecdysone	(McBrayer et al. 2007; Yamanaka, Rewitz, et al. 2013)
Phototaxis	DH44	Light avoidance	(Imambocus et al. 2025)
	DILP7	Light avoidance	(Imambocus et al. 2025)
	LK	Light avoidance	(Imambocus et al. 2022)
	MIP	Light avoidance	(Imambocus et al. 2022)
	PDF	Larval visual system (anatomy)	(Sorkaç et al. 2023; Sprecher et al. 2011)
	PTTH	Light avoidance	(Yamanaka, Romero, et al. 2013)
**Adult** ^b^			
Aggression (in males, unless specified)	DH44	Receptor analysis	(Y. K. Kim et al. 2018)
	DSK	IPCs or MP1/MP3 neurons	(P. Agrawal et al. 2020; J. Luo et al. 2014; Williams et al. 2014; F. Wu et al. 2020)
	Natalisin	Brain interneurons	(H. Jiang et al. 2013)
	NPF	Brain interneurons	(Dierick and Greenspan 2007)
	Sex peptide	In females	(Bath et al. 2017)
	TK	Brain interneurons	(Asahina et al. 2014; Wohl et al. 2023)
Courtship and reproduction	AstC	Clock neurons	(Meiselman, Alpert, et al. 2022)
	CRZ	Brain or VNC neurons	(Bergland et al. 2012; Bonheur et al. 2023; H. ‐H. Lin et al. 2022; Tayler et al. 2012; Zer‐Krispil et al. 2018b; N. Zhang et al. 2025; T. Zhang et al. 2024)
	DH44	MNCs or PC1 neurons	(K. ‐M. Lee et al. 2015) (X. Jiang et al. 2024; D. ‐H. Kim et al. 2024; H. Zhao et al. 2024)
	ILP2, 3, 5	IPCs	(S. ‐L. Chen et al. 2022; Lebreton et al. 2015; L. Zhang et al. 2022)
	DSK	IPCs or MP1/MP3 neurons	(Fedina et al. 2023; H. ‐F Li et al. 2024; S. Wu et al. 2019)
	ETH	Inka cells (peritracheal cells)	(Deshpande et al. 2019; S. S. Lee and Adams 2021; Meiselman et al. 2017; Meiselman, Ganguly, et al. 2022)
	LK	VNC neurons (ABLKs)	(C. Liu et al. 2020)
		ABLKs via PC1 neurons	(J. Chen et al. 2025)
	MIP	Interneurons VNC (females)	(Y. H. Jang et al. 2017; Shao et al. 2019)
	Natalisin	Brain interneurons	(H. Jiang et al. 2013)
	NPF	Brain interneurons or gut EECs	(Gendron et al. 2014; Hoshino et al. 2023; W. J. Kim et al. 2013; Ryvkin et al. 2024; Shohat‐Ophir et al. 2012)
	Orcokinin	Brain neurons	(Silva et al. 2021)
	PDF	Clock circuit	(Fujii and Amrein 2010; W. J. Kim et al. 2013; Krupp et al. 2013)
	Sex peptide	Females	(P. S. Chen et al. 1988; Kubli 2003; Wolfner 2002; C. H. Yang et al. 2009)
	SIFamide	Global brain interneurons	(Sellami and Veenstra 2015; Y. Song et al. 2024; Terhzaz et al. 2007; T. Zhang et al. 2024)
Locomotion^c^	sNPF, TK	Central complex (walking)	(Kahsai, Martin, and Winther 2010)
	FMRFamide	Brain, via dopamine (flight)	(Ravi et al. 2018)
	PDF	PDF receptor knockdown	(T. Agrawal et al. 2013)
Ovary function (maturation, ovulation, oviposition)	AKH	Corpora cardiaca	(E. H. Lee et al. 2026)
	CNMa	Fat body	(Grmai et al. 2024)
	DH44	Spermatheca (sperm release)	(K. ‐M. Lee et al. 2015)
	FMRFamide	VNC neurons (Va; egg laying)	(Sadanandappa et al. 2026)
	ILPs	IPCs (CA via JH)	(Grönke et al. 2010)
	ILP7	VNC efferents	(C. H. Yang et al. 2008)
	ILP8	Ovary follicle cells	(Volonté et al. 2026)
	NPF	Gut EECs	(Hoshino et al. 2023; E. H. Lee et al. 2026)
	Orcokinin	General knockdown	(Silva et al. 2021)
Diapause^d^ (in females)	AKH	Corpora cardiaca	(Kučerová et al. 2016)
	AstC	Clock neurons	(Meiselman, Alpert, et al. 2022)
	DH31	LNCs	(Kurogi et al. 2023)
	ILPs	IPCs	(Kubrak et al. 2014; Kurogi et al. 2021; Nagy et al. 2019)
	PDF	Clock neurons	(Hidalgo et al. 2023; Nagy et al. 2019; Ojima et al. 2018)
	sNPF	Clock neurons	(Kurogi et al. 2021; Nagy et al. 2019)

*Note:* Peptide roles in this table include actions on cells, tissues, behavior, and physiology (often given in very general terms), and some details are listed under specifics.

Peptide abbreviations in list of acronyms.

Other acronyms: IPCs, insulin‐producing cells; VNC, ventral nerve cord; PC1, sex‐specific neurons; MP1/MP3, DSK interneurons; EECs, enteroendocrine cells; CA, copora allata; JH, juvenile hormone.

^a^ Action and/or cells of interest (cells releasing NPH or target of NPH).

^b^ Although adult flies have a complex visual system and a number of neuropeptides have been localized to the optic lobes (Nässel, Zandawala, et al. 2019), there are no data on functional roles of NPHs except for PDF.

^c^ The roles of NPHs in adult locomotion, including flight, have not been explored in any detail.

^d^ Diapause in *Drosophila* is characterized by reproductive arrest with decreased activity and metabolism (Kubrak et al. 2014; Tatar and Yin 2001).

**TABLE 2:** Larval roles of neuropeptides and peptide hormones in *Drosophila*.

**Function**	**Neuropeptide (mechanism/role)** ^a^	**Cell system** ^b^	**References**
Nutrient sensing	AKH (glucose sensing)	APCs (CC)	(J. Kim and Neufeld 2015; S. K. Kim and Rulifson 2004)
	CRZ (fructose sensing)	LNC^CRZ^	(Kulshrestha et al. 2024; Mishra et al. 2013)
	DH31 (amino acid sensing)	Gut EECs	(J. H. Park et al. 2016)
	DH44 (glucose sensing)	MNC^DH44^	(Poe et al. 2024)
Olfaction/taste	Hugin‐PK (taste)	SEZ	(Hückesfeld et al. 2016; Schlegel et al. 2016)
	NPF (olfaction)	Brain neurons	(Rohwedder et al. 2015)
Learning	sNPF (olfactory learning)	Mushroom body	(Lyutova et al. 2019)
Food seach/feeding	AstA (foraging)		(C. Wang et al. 2012)
	CCHa2 (appetite)	Fat body	(Brunßen and Suter 2024; Sano 2015)
		Gut EECs	(Ren et al. 2015)
	CRZ (via sugar sensing)	LNC^CRZ^	(Kulshrestha et al. 2024; Mishra et al. 2013)
	Hugin‐PK (microbial stress related)	SEZ	(Surendran et al. 2017)
	Hugin‐PK	SEZ	(Hückesfeld et al. 2016; Melcher and Pankratz 2005; Schlegel et al. 2016; Schoofs et al. 2014)
	NPF (feeding decision)	Brain neurons	(de Tredern et al. 2025)
	NPF (food odor/appetite	Brain neurons	(Pu et al. 2018; Y. Wang et al. 2013)
	NPF (food search, olfaction)	Brain neurons	(Rohwedder et al. 2015)
	sNPF (feeding)	CNS neurons	(K. S. Lee et al. 2009; K. S. Lee et al. 2004)
Growth/development/metabolism	AstA	Brain neurons, Via PTTH and ILPs	(Barredo et al. 2021; Deveci et al. 2019)
	Bursicon (via muscle and IIS)	VNC neurons	(Kubrak et al. 2024)
	CCHa2 (via IPCs/IIS)	Fat body	(Brunßen and Suter 2024; Sano et al. 2015)
		Gut EECs	(Ren et al. 2015)
	CRZ (via PTTH and Ecd)	LNC^CRZ^	(Imura et al. 2020)
	CRZ (mechanism?)	LNC^CRZ^	(Megha et al. 2019)
	ILP2,3,5	IPC (MNC^ILP^)	(Brogiolo et al. 2001; Dunham and Bland 2026; Géminard et al. 2006; Grönke et al. 2010; Koyama and Mirth 2018; Okamoto and Mizoguchi 2026; Tennessen and Thummel 2011)
	ILP6	Fat body	(Okamoto et al. 2009; Slaidina et al. 2009)
	ILP7	VNC neurons, via ILPs (IPCs)	(Prince et al. 2021)
	ILP8	Imaginal discs	(Colombani et al. 2015; Colombani et al. 2012; Garelli et al. 2012; Garelli et al. 2015)
	Limostatin (Lst)	Gut EECs	(Bai et al. 2026)
	TK (via insulin signaling)	Brain (ICN)	(Meschi et al. 2019)
	sNPF (via insulin signaling)	CNS neurons	(K. S. Lee et al. 2009, 2004)
Locomotion	AKH	CC	(Ibrahim et al. 2018)
	Hugin‐PK (feeding‐locomotion)	SEZ	(Schoofs et al. 2014)
	LK (turning)	ABLK	(Okusawa et al. 2014)
	DSK	Receptor analysis	(X. Chen et al. 2012)
	Proctolin (crawling)	VNC	(Ormerod et al. 2016)
Nociception/stress/escape locomotion	AKH (UV stress)	APCs (CC)	(Guntur et al. 2015)
	Bursicon (stress/immune response)		(An et al. 2012)
	CRZ (via nutrient sensing)	CN neurons	(Nakamizo‐Dojo et al. 2023)
	DH44 (light avoidance; nutrient‐dependent)	MNC^DH44^ Via ILP7 neurons and ABLK	(Imambocus et al. 2025)
	DILP7 (light avoidance)	DP neurons (VNC)	(Imambocus et al. 2025)
	DSK (heat nociception)	Brain descending neurons	(Oikawa et al. 2023)
	LK (light avoidance; nutrient‐dependent)	ABLK	(Imambocus et al. 2025; Imambocus et al. 2022)
	LK (nociception)	ABLK	(K. Li et al. 2023)
	MIP (AstB; light avoidance)	VNC Via ILP7 neurons and ABLK	(Imambocus et al. 2022)
	sNPF (mechanosens.)	ILP7 neurons (DP) VNC	(C. Hu et al. 2017)
	TK (heat nociception)	Brain neurons	(Gu et al. 2022; Im et al. 2015)
Ca^2^ ^+^ mobilization	CAPA	Renal tubules	(Okamoto et al. 2026)
Gut peristalsis	DH31	Gut EECs	(LaJeunesse et al. 2010)
Osmoregulation	ITP	Hindgut	(Watanabe et al. 2026)
Defecation	PDF	VNC	(W. Zhang et al. 2014)
Immune response	DH31 (via IMD path.)	Gut EECs	(Tleiss et al. 2024)
	Hugin‐PK	SEZ neurons	(Surendran et al. 2017)
Phototaxis	LK (light avoidance)	ABLK	(Imambocus et al. 2025)
	PDF (larval visual system)	sLNv (brain)	(Sprecher et al. 2011)
	PTTH (light avoidance)	LNC^PTTH^	(Yamanaka, Romero, et al. 2013)
Activity/sleep	DH44 (nutrient‐dependent)	MNC^DH44^ (DN1 inputs)	(Poe et al. 2023; Poe et al. 2024)
Regulation hormone Release	AKH (ILP release)	APCs (CC)	(Kim 2004) #1670
	CCHa2 (IPCs; ILP2, 5 release)	Fat body	(Sano et al. 2015)
	CRZ (PTTH and Ecd)	LNC^CRZ^	(Imura et al. 2020)
	Hugin‐PK (CA, but not JH)	SEZ	(Mizuno et al. 2021)
	NPF (ecdysone regulation)	EECs?	(Kannangara et al. 2020)
Molting/ecdysis	AstA (timing of growth and ecdysis)	Brain neurons, Via PTTH and ILPs	(Deveci et al. 2019)
	AstC (metamorphosis)	LNC^AstC^	(C. Li et al. 2024)
	Bursicon (ecdysis cascade)	VNC	(Diao et al. 2017)
	Bursicon (ecdysis stress)	VNC	(An et al. 2012)
	CCAP (ecdysis cascade)	VNC	(J. H. Park et al. 2003)
	EH	Brain	(McNabb et al. 1997; Scott et al. 2020)
	ETH	Inka cells	(Y. ‐J. Kim et al. 2006; B. H. White and Ewer 2014; Žitňan et al. 2007; Zitnan et al. 1996)
	FMRFamide	VNC	(Y. ‐J. Kim et al. 2006)
	Hugin‐PK	SEZ	(Meng et al. 2002)
	LK (pre‐ecdysis)	ABLK	(D. H. Kim et al. 2015; Y. ‐J. Kim et al. 2006)
	LK (tracheal clearing)	ABLK	(D. H. Kim et al. 2018)
	MIP (glue expulsion)	VNC	(Fernandez‐Acosta et al. 2025)
	Myosuppressin (DMS)	Not specified	(Ruf et al. 2017)
	PDF (eclosion)	PDF‐Tri (brain)	(Selcho et al. 2018)
	PTTH	LNC^PTTH^	(McBrayer et al. 2007; Yamanaka, Rewitz, et al. 2013)
Development, other	CRZ	LNC^CRZ^	(Barredo et al. 2021; Megha et al. 2019)
	GPA2/GPB5; LGR1 (larval to pupal trans)	VNC	(Vandersmissen et al. 2014)
	MIP (glue expulsion)	VNC	(Fernandez‐Acosta et al. 2025)
	NPF (ecdysone regul.)	EECs?	(Kannangara et al. 2020)
	sNPF (mechanism?)	LNC^CRZ^	(Megha et al. 2019)

Notes to Table

^a^ a Note that some actions are indirect and some are classified very generally.

^b^ b Some acronyms are designations of neurons.

^c^ c Specific cells in circuit not known.

Peptide abbreviations in list of acronyms.

*Other acronyms:* APCs, AKH‐producing cells; CC, corpora cardiaca; EEC, enteroendocrine cells; IPCs, insulin‐producing cells; LNCs, lateral neurosecretory cells; MNCs, median neurosecretory cells; NSC, neurosecretory cell; SEZ, subesophageal zone; VNC, ventral nerve cord.

### 2.2 Adult

Life of the adult fly is complex and dynamic. Having wings and legs, the fly is highly mobile and can move long distances for food, partners, and egg‐laying sites. The feeding habits and diet differ from those of the growing larva. The sensory input of the adult has expanded with additional odors and tastes, including sex pheromones, and importantly the elaborate compound eyes allow advanced vision. The circuitry in the brain constituting the biological clock is far more complex in adults, and it times most aspects of physiology and behavior, including sleep and reproduction (see recent reviews Reinhard et al. 2024; Wegener et al. 2026). Several additional behaviors are hallmarks of the adult reproductive insect, such as flight navigation, courtship, aggression, and oviposition. On the other hand, food intake, digestion, and metabolism, as well as nutrient and energy homeostasis and regulation of water and ion balance, are requirements both in larvae and adults. However, combined with sexual maturation, more varied behavior (including reproduction), and longer lifespan, the adult requires more complex regulatory systems. Importantly, the adult brain in *Drosophila* contains sexually dimorphic circuits that control social behavior (e.g., mating, reproduction, and aggression), and some of this signaling is peptidergic (Auer and Benton 2016; Dickson 2008; Kim and Pan 2026; Pavlou and Goodwin 2013). Numerous studies have addressed the involvement of NPHs in adult behavior and physiology (summarized in recent reviews; Koyama et al. 2023; Nässel and Zandawala 2019, 2022; Schoofs et al. 2017), and adult NPH functions are listed in Tables 1, 2, 3. Curiously, there are insects (e.g., aphagous coleoptera and Lepidoptera, including the model insect *Bombyx mori*) that do not feed as adults (but use resources stored by the larva) and display a shorter lifespan as adults than as larvae (see, e.g., R. B. Davis et al. 2016; Jervis et al. 2005; Paukku and Kotiaho 2005). Probably some of the adult NPH signaling systems in such insects differ from those in feeding insects. However, peptidomics indicate that the number of NPHs in larval and adult brains in *B. mori* does not differ much (X. Liu et al. 2012).

**TABLE 3:** Neuropeptides and peptide hormones that regulate behavior in adult *Drosophila*.

Role^a^	Neuropeptide	Circuit/neurons^b^	References
Olfaction	CCHa1	OSNs Antennal lobe (AL)	(Farhan et al. 2013)
	DILPs	OSN‐PN (AL)	(Root et al. 2011)
		OSN (VM2 in AL)	(Lebreton et al. 2015)
	ETH	Inka cells to OR	(Meiselman, Ganguly, et al. 2022)
	Hugin‐PK	SEZ‐NSC via SIFa neurons to ORs	(Martelli et al. 2017)
	MIP	OSN‐PN (AL)	(Hussain et al. 2016)
		LN‐PN (AL)	(Sizemore et al. 2023)(Sizemore et al. 2022)
		Via SIFa neurons	(Martelli et al. 2017)
	NPF	Brain interneurons	(Beshel and Zhong 2013; S. Lee et al. 2017)
	SIFa	Brain interneurons to ORs	(Martelli et al. 2017)
	sNPF	OSN‐PN (AL)	(Root et al. 2011)
	TK	LN‐OSN‐PN (AL)	(French et al. 2021; Ignell et al. 2009; Ko et al. 2015)
Taste	AKH	Gr5a	(Bharucha et al. 2008; Jourjine et al. 2016; Yu et al. 2016)
	DSK	MP1/MP3‐Gr64f	(D. Guo et al. 2021)
	LK	SELK neurons (SEZ)	(Chu et al. 2024; López‐Arias et al. 2011; Mollá‐Albaladejo et al. 2025; Savaş et al. 2026)
	sNPF	LNCs‐Gr66a^c^	(Inagaki et al. 2014; W. J. Kim et al. 2013)
	TK	Pheromone pathway	(Shankar et al. 2015)
		Central complex and Gr43a (fructose)	(Musso et al. 2021)
Food search/feeding	AKH	CC cells	(Bharucha et al. 2008; Gáliková et al. 2015; G. Lee and Park 2004; Pauls et al. 2021; Yu et al. 2016)
	AstA	Brain neurons or EECs	(J. Chen et al. 2016; Hentze et al. 2015; Hergarden et al. 2012; Tsao et al. 2018)
	AstC	EECs	(Kubrak et al. 2022)
		Brain neurons	(H. ‐H. Lin et al. 2022)
	Bursicon	EECs	(Hadjieconomou et al. 2020)
	CAPA	NSCs brain, VNC	(Koyama et al. 2021)
	CCAP	Brain interneurons	(González Segarra et al. 2023; Williams et al. 2020)
	CCHa2	Gut, fat body	(Ren et al. 2015)
	CNMa	Gut enterocytes	(B. Kim et al. 2021)
	CRZ	LNCs	(Kubrak et al. 2016)
	DH31	EECs	(H. ‐H. Lin et al. 2022)
	DH44	MNCs and VNC neurons	(Dhakal et al. 2022; Dus et al. 2015; Kasturacharya et al. 2023; Oh et al. 2021; Z. Yang et al. 2018; Zandawala et al. 2018)
	DILP2, 3, 5	IPCs	(Nässel and Vanden Broeck 2016; Owusu‐Ansah and Perrimon 2014) (Barber et al. 2021; González Segarra et al. 2023; X. Li et al. 2024; P. Wang et al. 2020)
	DILP8	Tumor cells to brain neurons	(Yeom et al. 2021)
	DMS	MNCs	(Hadjieconomou et al. 2020)
		Brain neurons	(G. Wu et al. 2024)
	DSK	IPCs, MP1/MP3	(D. Guo et al. 2021; X. Li et al. 2024; Nässel and Wu 2022; Söderberg et al. 2012)
	FMRFa	Brain neurons	(G. Wu et al. 2024)
		VNC‐NSCs to GRs	(T. Song et al. 2023)
	Hugin	SEZ neurons	(King et al. 2017; Martelli et al. 2017)
	ITP	LNCs	(Galikova et al. 2018; Gera et al. 2025)
	LK	Brain neurons	(Al‐Anzi et al. 2010; Zandawala et al. 2018)
	MIP/AstB	Brain neurons	(Min et al. 2016)
	NPF	Brain neurons	(Beshel et al. 2017; Chung et al. 2017; Corfas et al. 2019; Landayan et al. 2021; S. Lin et al. 2019; Tsao et al. 2018)
		Gut EECs	(Gao et al. 2024)
	RYa	Not clear	(Ida et al. 2011)
	Sex peptide	Via sperm, female response	(Barnes et al. 2008; Carvalho et al. 2006; Chapman et al. 2003; Kubli 2003; Ribeiro and Dickson 2010)
	SIFa	Global interneurons	(Dreyer et al. 2019; Martelli et al. 2017; W. Zhang et al. 2024)
	sNPF	OSNs‐PNs (AL), local brain interneurons, MB circuits	(de Tredern et al. 2025; Hong et al. 2012; Root et al. 2011; Tsao et al. 2018)
		LNCs (female diet)	(C. Liu et al. 2024)
	TK	OSNs‐PNs (AL)	(Ko et al. 2015)
		SMP‐TK	(Qi et al. 2021)
		Central complex (FB)	(Musso et al. 2021)
Thirst/drinking	AstA	JanuAstA (SEZ)	(Landayan et al. 2021)
	CCAP	Brain neurons	(González Segarra et al. 2023; Gonzalez‐Suarez and Nitabach 2018)
	CCHa2	CCHa2‐R neurons (SEZ)	(González Segarra et al. 2023; Gonzalez‐Suarez and Nitabach 2018)
	DILPs	IPCs	(González Segarra et al. 2023)
	LK	Brain neurons	(Chu et al. 2024)
	NPF	Brain AstA‐R neurons	(Landayan et al. 2021)
	ITP	LNC^ITP^ (ALK)	(Galikova et al. 2018)
	Ast‐A	Larvae	(C. Wang et al. 2012)
	DH44	Brain neurons Not MNCs	(X. Jiang et al. 2024; H. Zhao et al. 2024)
	DSK	MP1/MP3	(X. Chen et al. 2012; S. Wu et al. 2019)
	Hugin‐PK	SEZ Hugin cells	(King et al. 2017; Schlegel et al. 2016)
	PDF	Central complex	(Pírez et al. 2013)
	TK	Brain neurons	(H. Zhao et al. 2024)
Explorative walking	sNPF	Central complex	(Kahsai, Martin, and Winther 2010)
	TK	Central complex	(Kahsai, Martin, and Winther 2010)
Clock/circadian activity/sleep	AstA	Clock circuit	(Reinhard et al. 2022)
	AstC	Clock circuit	(Díaz et al. 2019)
	CCHa1	Clock circuit	(Fujiwara et al. 2018; Gopalakrishnan et al. 2026; Kuwano et al. 2023)
	CNMamide	Clock circuit	(X. Jin et al. 2021)
	DH31	Clock circuit	(Chong et al. 2024; Goda et al. 2016; Kunst et al. 2014)
	ITP	Clock circuit	(Hermann‐Luibl et al. 2014; Le et al. 2024)
	NPF	Clock circuit	(Chung et al. 2017; Hamasaka et al. 2010; Hermann et al. 2012)
	PDF	Clock circuit	(Liang et al. 2017; Renn et al. 1999)
	sNPF	Clock circuit	(Liang et al. 2017; Shang et al. 2013)
	Sparkly	Clock circuit	(Sukumar et al. 2024)
	Trissin	Clock circuit	(Sekiguchi et al. 2024)
Activity/sleep (outside clock)	AKH	CC (APCs) via OA and FB	(Pauls et al. 2021)
	AstA	CNS neurons	(J. Chen et al. 2016; Dissel et al. 2022; Donlea et al. 2018; Ni et al. 2019)
	Bursicon	CNS neurons	(Cavey et al. 2016; Melnattur et al. 2019)
	CCHa1	Gut EECs	(Gopalakrishnan et al. 2026; Titos et al. 2023)
	CCHa2	Fat body via IPCs	(Biglari et al. 2025)
	Corazonin	Brain neurons	(Afonso et al. 2015)
	DH31	Central complex	(Lyu et al. 2023)
		Via MNCs	(Chong et al. 2024)
	DH44	MNCs	(Barber et al. 2021; Cavanaugh et al. 2014; King et al. 2017; Poe et al. 2024)
	DILPs	IPCs	(Barber et al. 2021; Yamaguchi et al. 2022)
	FMRFa	CNS neurons	(Lenz et al. 2015)
	Hugin‐PK	SEZ neurons	(King et al. 2017; Schwarz et al. 2021)
	LK	Lateral horn neurons (LHLK)	(Cavey et al. 2016; Murakami et al. 2016; Murphy et al. 2016; Yurgel et al. 2019)
	MIP	Brain neurons	(Oh et al. 2014)
		ICLI neurons (brain)	(Petzold and Gilestro 2024)
	Sex peptide	In females	(Isaac et al. 2010)
	SIFa	Global interneurons	(Cavanaugh et al. 2014; Cavey et al. 2016; Huang et al. 2021; S. Park et al. 2014)
	sNPF	MB neurons	(W. F. Chen et al. 2013)
	TK	Fan‐shaped body	(French et al. 2021; S. H. Lee and Kim 2021)
		Brain interneurons	(S. H. Lee et al. 2021)
Aggression^d^	DH44	DH44‐R1 cells^e^	(Y. K. Kim et al. 2018)
	DSK	IPCs	(P. Agrawal et al. 2020; J. Luo et al. 2014; Williams et al. 2014)
		MP1/MP3 brain	(F. Wu et al. 2020)
	Natalisin	Brain interneurons	(H. Jiang et al. 2013)
	NPF	Brain interneurons	(Dierick and Greenspan 2007)
	Sex peptide	In females	(Bath et al. 2017)
	TK	LPP1B brain interneurons	(Asahina et al. 2014; Wohl et al. 2023)
Learning	AKH	CC (APCs) via OA	(Meschi et al. 2024)
	AstA	Brain neurons	(Yamagata et al. 2016)
	CCHa2	Brain neurons	(Yamagata et al. 2022)
	Corazonin	Brain neurons^f^	(Zer‐Krispil et al. 2018b)
	DH31	Clock neurons	(Frantzmann et al. 2023)
		OA neurons	(Lyu et al. 2023)
	DILPs	IPCs	(Chambers et al. 2015; Naganos et al. 2012; Tanabe et al. 2017)
	ETH	Inka cells	(S. H. Lee et al. 2021)
	LK	Brain neurons	(Senapati et al. 2019)
	NPF	Brain neurons	(Krashes et al. 2009; Rohwedder et al. 2015; Shao et al. 2017)
	SIFa	Brain neurons downstream clock	(Y. Zhang et al. 2021)
	sNPF	Mushroom Body	(Knapek et al. 2013)
	PDF	Clock neurons	(Inami et al. 2022)
Nociception	AstC	AstC receptor, EPI neurons	(Bachtel et al. 2018; J. Liu et al. 2023)
	Corazonin	Brain neurons (L3)	(Nakamizo‐Dojo et al. 2023)
	LK	LK receptor	(Ohashi and Sakai 2018)
	TK	Brain neurons (adult); visual aversion	(Gu et al. 2022; Im et al. 2015; Tsuji et al. 2023)
Ethanol‐related behaviors	Corazonin	LNCs	(McClure and Heberlein 2013; Sha et al. 2014)
	DILPs	IPCs	(Corl et al. 2005)
	NPF	Brain interneurons	(Shohat‐Ophir et al. 2012)
Courtship and reproduction	AKH	Corpora cardiaca (pheromone production and perception)	(Lebreton et al. 2016)
	AstC	Clock neurons	(Meiselman, Alpert, et al. 2022)
	Corazonin	VNC neurons (males)	(Tayler et al. 2012; Zer‐Krispil et al. 2018a)
		Brain neurons	(Bergland et al. 2012; Bonheur et al. 2023; H. ‐H. Lin et al. 2022; T. Zhang et al. 2024)
	DH44	MNCs	(K. ‐M. Lee et al. 2015)
		PC1 neurons	(X. Jiang et al. 2024; D. ‐H. Kim et al. 2024; H. Zhao et al. 2024)
		Thoracic histamine neurons (MtAHN)	(Bennett et al. 2026)
	DILPs	IPCs	(S. ‐L. Chen et al. 2022; Lebreton et al. 2015; L. Zhang et al. 2022)
	DSK	MP1/MP3	(X. Li et al. 2024; S. Wu et al. 2019)
		IPCs	(Fedina et al. 2023)
	ETH	Inka cells to JH	(Meiselman et al. 2017)
		Inka cells to OSNs and GRNs	(Deshpande et al. 2019; Meiselman, Ganguly, et al. 2022)
		Inka cells to MB	(S. S. Lee and Adams 2021)
	LK	VNC neurons	(J. Chen et al. 2025; C. Liu et al. 2020)
	MIP/AstB	Interneurons VNC (females)	(Y. H. Jang et al. 2017; Shao et al. 2019)
	Natalisin	Brain interneurons	(H. Jiang et al. 2013)
	NPF	Brain interneurons	(Gendron et al. 2014; W. J. Kim et al. 2013; Ryvkin et al. 2024; Shohat‐Ophir et al. 2012)
		Gut EECs (oogenesis)	(Hoshino et al. 2023)
	Orcokinin	Brain neurons	(Silva et al. 2021)
	PDF	Clock circuit	(Fujii and Amrein 2010; W. J. Kim et al. 2013; Krupp et al. 2013)
	Sex peptide	Females (via semen)	(P. S. Chen et al. 1988; Kubli 2003; Wolfner 2002; C. H. Yang et al. 2009)
	SIFa	Global interneurons	(Sellami and Veenstra 2015; Terhzaz et al. 2007; T. Zhang et al. 2024)
Sperm storage	DH44	In females	(K. ‐M. Lee et al. 2015)
Oviposition	DILP7	VNC efferents	(C. H. Yang et al. 2008)
	FMRFamide	VNC(?)	(Sadanandappa et al. 2026)
	Orcokinin	Gene knockdown	(Silva et al. 2021)

Notes to Table

^a^ Note that some actions are indirect and some are classified very generally

^b^ Some acronyms are designations of neurons.

^c^ The LNCs regulate Gr66 bitter taste with sNPF.

^d^ Males if not indicated.

^e^ Specific cells in circuit not known.

^f^ A successful copulation is a reward in male flies and strengthens long‐term appetitive memories.

Peptide abbreviations in list of acronyms.

Other acronyms: AL, antennal lobe; APCs, AKH‐producing cells; CC, corpora cardiaca; EEC, enteroendocrine cells; GR, Gr5a, Gr43a, Gr64f and Gr66a, gustatory receptors; IPCs, insulin‐producing cells; JanuAstA, AstA‐expressing interneurons; LN, local neurons; LNCs, lateral neurosecretory cells; MB, mushroom bodies; MNCs, median neurosecretory cells; MP1/MP3, DSK‐expressing interneurons, NSC, neurosecretory cell; OR, olfactory receptor; OSN, olfactory sensory neurons; PN, projection neurons; SEZ, subesophageal zone; SMP‐TK, TAG, thoracico‐abdominal ganglion; TK‐expressing interneurons; VM2, pheromone‐sensitive glomerulus in antennal lobe; VNC, ventral nerve cord.

This table is updated and altered from Nässel, Pauls, and Huetteroth (2019) and Nässel and Zandawala (2019).

## 3 NPHs in Larvae and Adults, a Brief Overview

In *Drosophila*, more than 50 genes have been identified that code for precursor proteins from which NPHs can be processed (reviewed in Nässel and Zandawala 2019). Most of these NPHs act on G‐protein coupled receptors (GPCRs), but some act on tyrosine kinase receptors (TKR) or membrane guanylate cyclase receptors (mGCs; Nässel and Zandawala 2019). With a few exceptions, the same set of NPHs and receptors have been identified in larvae and adults. The NSCs‐producing eclosion hormone (EH) and prothoracicotropic hormone (PTTH) are specific to development and can no longer be found in the mature adult insects (Horodyski et al. 1993; McBrayer et al. 2007) although a set of novel non‐canonical EH interneurons appear in pharate adults (Scott et al. 2020). A feature specific to adult *Drosophila* is a small set of bioactive peptides produced in the male accessory glands (and not found in larvae), such as sex peptide, Acp26Aa (ovulin), and Dup99B (P. S. Chen et al. 1988; Kubli 2003; Wolfner 2002).

Importantly, there are major differences between larval and adult NPH functions or actions, linked to stage‐specific physiology and behavior. Thus, in the larvae, many NPHs are critical in regulating growth and development, including molting/eclosion. On the other hand, adult‐specific behavior and physiology in *Drosophila* means that many NPHs play new key roles in, for instance, social behavior, locomotion, reproduction, and adult diapause (in *Drosophila*, reproductive diapause is a reversible dormancy; Kubrak et al. 2014; Tatar and Yin 2001). Moreover, the remodeling of neurons during metamorphosis leads to substantial alterations of the synaptic connectome, and as a consequence interneuronal neuropeptides may act locally as neuromodulators in novel circuits (with associated novel functions). Note that NPHs also play roles during embryonic and pupal stages, but apart from the involvement in adult eclosion, these are not discussed here.

A reductionist view of NPH signaling specific to larvae and adults, respectively, is provided in Figure 1. This figure shows NPHs that regulate larval‐specific processes in growth, ecdysis, and control of developmental hormones (e.g., juvenile hormone [JH] and ecdysone) and other aspects of development. The adult‐specific NPH functions are in aggression, courtship and other reproductive behavior, ovary development (sexual maturation), and reproductive diapause. As seen in Figure 1, the number of NPHs that are involved in either larval or adult‐specific functions is quite high. Note that some NPHs display both larval‐ and adult‐ specific functions (i.e., switch of functions), and importantly many of the same NPHs play other roles that are not stage‐specific (see Tables 2 and 3). As an example of the former, allatostatin C (AstC) and myoinhibitory peptide (MIP) play roles in molting/ecdysis as well as in courtship and reproduction. Furthermore, a few NPHs play similar roles in larvae and adults. For instance, in food seeking and feeding, several NPHs have been identified in both stages (although detailed mechanisms may differ); both growth and reproductive physiology are nutrient‐dependent. These are adipokinetic hormone (AKH), allatostatin A (AstA), CCHamide2 (CCHa2), corazonin (CRZ), diuretic hormone 44 (DH44), DH31, Hugin‐PK, NPF, and sNPF (see Table 1), produced by NSCs, other neurons, or gut EECs. As seen in Table 2, the number of NPHs that have been implicated in foraging and feeding in larvae is quite a bit smaller than the NPHs identified in adults. This probably reflects a bias due to a higher number of studies of adults rather than the correct number of larval NPHs involved. In contrast, the studies of NPHs in nociception are more numerous in larvae (11 NPHs) than adults (4 NPHs), and only CRZ, leucokinin (LK), and tachykinin (TK) play this role in both stages (Tables 2 and 3). A more comprehensive comparison between larval‐ and adult‐specific roles of NPHs is shown in Table 1.

**FIGURE 1:**
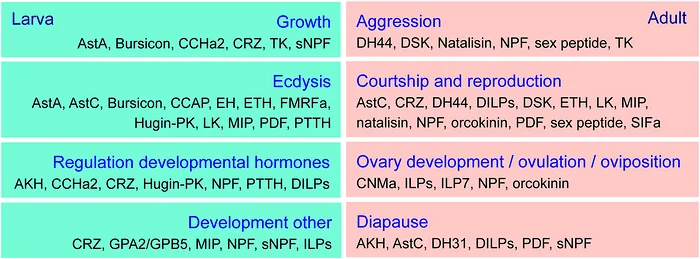
Neuropeptides regulating functions specific to larval versus adult *Drosophila*. These eight functions or actions are broadly defined and were selected since they are clear‐cut stage‐specific. Note that the NPHs listed play roles additional to the ones given here in both stages (for details and references, see Tables 2, 3, 4). For acronyms, see the list of peptide acronyms.

How does the number of peptidergic neurons change between the larval and adult CNS? In general, the peptidergic neurons are more numerous in the adult stage, but there are some exceptions. For instance, the number of peptidergic NSCs in the brain is nearly the same in larvae and adults (Figure 2), as demonstrated in more detail in the next section. Also, the number of the globally arborizing SIFamide neurons in pars intercerebralis (where the median NSCs, MNCs, are located) remains at four (Terhzaz et al. 2007). A similar similarity between the stages is seen in the number of NSCs in the VNC as will also be shown later. In the next sections, I discuss different sets of peptidergic neurons in larvae and adults, starting with brain NSCs, thereafter NSCs of the VNC, followed by median peptidergic efferent neurons posteriorly in the abdominal neuromeres of the VNC that innervate peripheral targets.

**FIGURE 2:**
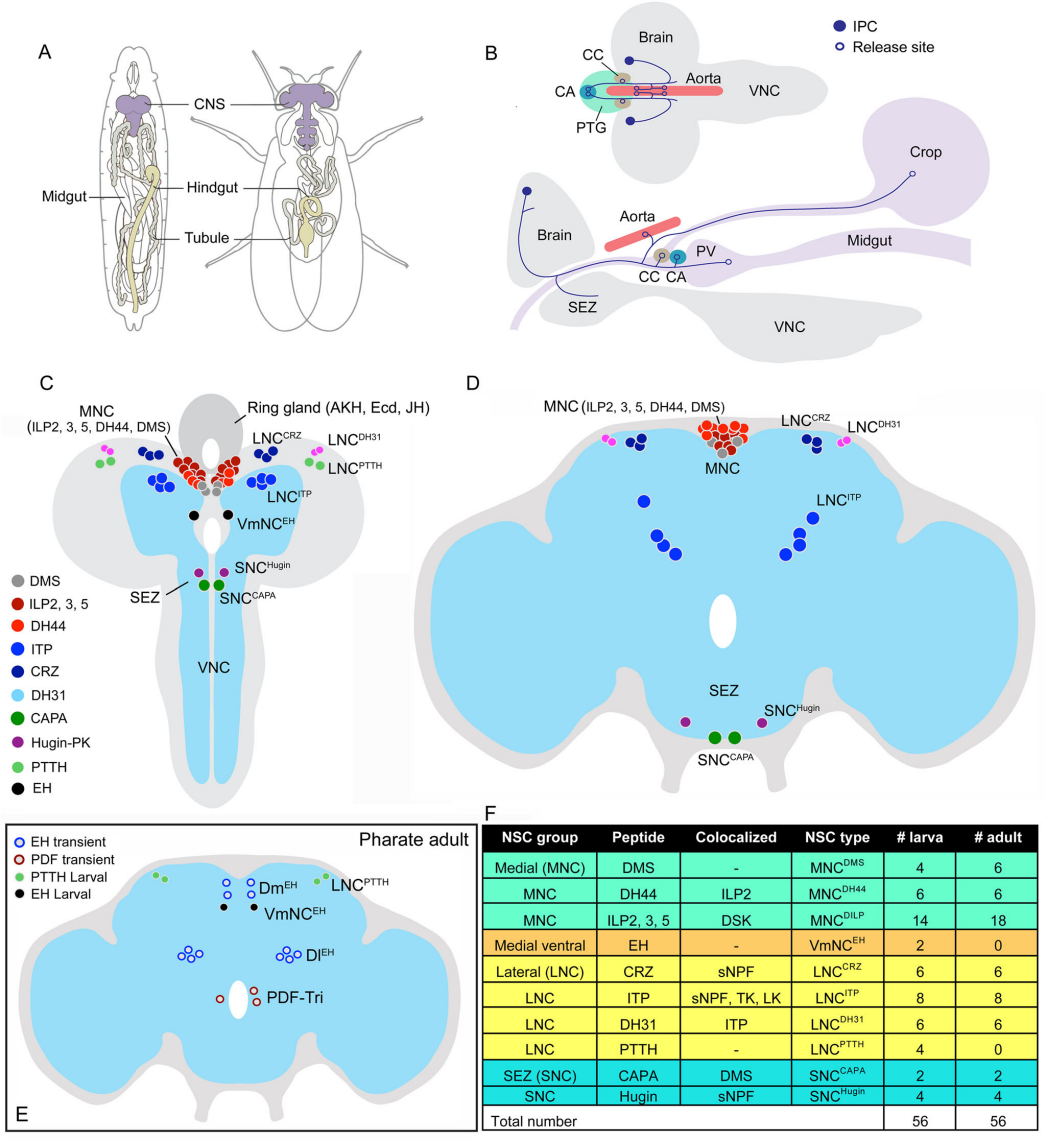
Neurosecretory cells (NSCs) in the brain of the larva and adult. (A) Overview. (B) The cerebral neuroendocrine system of larva (top; horizontal view) and adult (bottom; sagittal view). Insulin‐producing cells (IPC) of the median NSC group are used as an example. The IPCs have axon terminations (presumed release sites) in corpora allata (CA), corpora cardiaca (CC), and aorta in both stages. In adults, additional sites are found in the anterior midgut and crop. SEZ, subesophageal zone; VNC, ventral nerve cord. Note that in the larva the CC and CA are situated anteriorly in a ring‐shaped tissue called the ring gland (also containing the prothoracic gland, PTG), and in adults CC and CA are located in the anterior thorax, associated with the aorta and proventriculus (PV) of the gut. The lower scheme is redrawn from Nässel et al. (2013); the upper one is novel. (C) In the larval brain and subesophageal zone (SEZ), there are 56 peptidergic NSCs of 11 main types. In addition, there are 16 endocrine cells in the CC portion of the ring gland‐producing adipokinetic hormone (AKH; not shown). Further hormones (non‐peptidergic) are produced in the ring gland: juvenile hormone (JH) in the CA and ecdysone (Ecd) in the prothoracic gland. The brain NSCs express the following peptides: myosuppressin (DMS), insulin‐like peptide (DILP), diuretic hormone 31 (DH31), diuretic hormone 44 (DH44), ion transport peptide (ITP), corazonin (CRZ), capability (CAPA), Hugin pyrokinin (Hugin‐PK), prothoracicotropic hormone (PTTH), eclosion hormone (EH). The median NSCs (MNC) produce DILPs, DH44, and DMS. Other acronyms: LNC, lateral NSCs; VmNC, ventromedial NSCs; SNC, subesophageal NSCs. (D) In the adult brain, there are also 56 NSCs but of nine different types (see also 2F). The two EH and four PTTH‐producing NSCs are lost just after metamorphosis. There appear to be 18 DILP‐producing MNCs (IPCs) instead of the larval 14. The corpora cardiaca and its AKH‐producing cells are not shown here. (E) Some NSCs and other neurons are present in pharate adults and first days of adult life. Two sets are larval NSCs that survive metamorphosis, LNC^PTTH^ and VmNC^EH^ (McBrayer et al. 2007). The others DM^EH^, DI^EH^, and PDF‐Tri are peptidergic interneurons that produce EH and PDF and appear during metamorphosis but then undergo apoptosis in the adult (Horodyski et al. 1993; McNabb et al. 1997; Scott et al. 2020; Selcho et al. 2018). The NSC terminology and numerical data are based on (McKim et al. 2024). (F) Table providing details on the number of NSCs and their NPH expression. The primary peptides listed are the ones first discovered in the cells listed. The NPH colocalization shown is demonstrated in adults. This table is based on data from McKim et al. (2024).

## 4 Brain NSCs in the Larval and Adult Brain and Their Functions

A comparison between larval and adult NSC systems is a good starting point when looking at metamorphosis of NPH functions since basically all the NSCs and the NPHs they produce are found in both stages (Figure 2). The larval and adult CNS and brain NSC system are shown in Figure 2A,B. The larval NSCs (exemplified by insulin‐producing cells [IPCs] send axons that terminate in corpora cardiaca [CC] and corpora allata [CA] located in the ring gland and the anterior aorta, Figure 2B). In adults, the IPCs target CC, CA, the aorta, the proventriculus, and the crop (Figure 2B). A total of 12 different main NPHs are found in brain NSCs of larvae and 10 in those of adults; and some additional colocalized NPHs are detected in several of the NSCs (Tables 5 and 6; McKim et al. 2024). There are 10 major types of brain NSCs with identified NPHs in larvae (Figure 2C, Table 5), and eight of these types are also found in adults (Figure 2D, Table 6). Two sets of NSC cannot be detected in mature adults (although present in pharate adults): the EH‐producing Vm cells (also known as NSC^EH^) and the PTTH‐producing LNCs designated PG‐LP or l‐NSC^PTTH^ (Horodyski et al. 1993; McBrayer et al. 2007; McKim et al. 2024). These cells are however present in pharate adults and the first 1–2 days of adult life (Figure 2E). Interestingly, there are some neurons that can be found transiently in the brain of pharate adults (not in larvae; Figure 2E). These are not NSCs but interneurons that express EH and pigment‐dispersing factor (PDF) and are together with the other PTTH and EH neurons involved in regulation of ecdysis (Scott et al. 2020; Selcho et al. 2018). The number of cells within each of the eight NSC types found in larvae and adults is the same, except for a set of MNCs‐producing myosuppressin (DMS) and another set of MNCs‐producing insulin‐like peptides (ILPs; McKim et al. 2024; Ohhara et al. 2018; Figure 2F). The former cell type (SP3 or m‐NSC^DMS^) increases from four to six cells, and the latter (IPCs or m‐NSC^DILP^) increases from 14 to about 18 (but is variable). However, further analysis is required to clarify these increased numbers, especially since it was observed that there are 10 additional adult‐specific MNCs for which a NPH has not yet been identified (McKim et al. 2024). There are also 14 additional LNCs in adults with an unknown complement of NPHs (McKim et al. 2024). In the following and in Tables 5 and 6, the IPCs are treated as if they all co‐express the three ILPs. Of note is that the IPCs also express ILP1 during the pupal stage and the first 4–5 days of adult life (Y. Liu, Liao, et al. 2016). Hence, there is no ILP1 expression in IPCs in the feeding larval stages and adult feeding/reproductive stage, but interestingly ILP1 is upregulated during reproductive diapause in female flies (Y. Liu, Liao, et al. 2016) when feeding is reduced and reproduction arrested (Kubrak et al. 2014). The axonal projections and hormonal release sites (axon terminations) of LNCs and MNCs are similar in larvae and adults Figure 2B.

If we look into the functional roles (or actions in some cases) of NSCs and the NPHs they produce before and after metamorphosis, an interesting picture emerges. Few NPH functions are the same in the two stages (except some roles in feeding and metabolism). One caveat here is that the number of studies of larval NPH functions related to brain NSCs other than in development and growth is comparatively small, whereas in adults, numerous studies analyzed behavior and physiology (see Tables 2, 3, 4). The functions of larval and adult NPH produced by brain NSCs are summarized in Tables 5 and 6. Note that in the following, I refer primarily to these tables for cited work since references are so numerous.

**TABLE 4:** Neuropeptides and peptide hormones regulating physiology and homeostasis in adult *Drosophila*.

**Role**	**Peptide**	**Cells**	**Action**	**References**
Metabolism	AKH	CC (APCs)	Via fat body	(Braco et al. 2022; Gáliková et al. 2015; Nelson et al. 2021; Wat et al. 2021)
	AstA	Gut EECs	Via AKH to FB	(Y. Li et al. 2023)
	AstC	Gut EECs	Via AKH	(Kubrak et al. 2022)
	Bursicon	Gut EECs	Via AKH	(Scopelliti et al. 2014a)
	CAPA	VNC (and SEZ)	Via AKH	(Koyama et al. 2021)
	CCHa2	CCHa2 neurons	Diet restriction effect on lifespan	(K. Jin et al. 2020)
	Corazonin	Brain LNCs	Via fat body and via CAPA neurons	(Kubrak et al. 2016; Zandawala et al. 2021)
			Via ILPs and AKH	(Oh et al. 2019)
			Glycogen levels	(Patel and DiAngelo 2026)
	DH44	Brain MNCs	Lipid metabolism	(Nath et al. 2025)
	DILPs^a^	IPCs	Fat body (and indirect actions)	(Géminard et al. 2006; S. K. Kim et al. 2021; Nässel and Vanden Broeck 2016; Owusu‐Ansah and Perrimon 2014; Teleman 2010)
	DMS	Brain neurons	Via dopaminergic neurons	Wu (2024) #3406}
	FMRFa	Tv neurons (VNC)	Muscle glycogen	(Miyamoto et al. 2024)
		Brain neurons	To GRs (food intake)	(T. Song et al. 2023)
		Brain neurons	Via dopaminergic neurons	(G. Wu et al. 2024)
		VNC neurons?	To fat body	(T. Song et al. 2023)
	ITP	LNCs	Via AKH signaling	(Gáliková and Klepsatel 2022)
			Via fat body	(Gera et al. 2025)
	Limostatin	Corpora cardiaca	Via IPCs	(Alfa et al. 2015)
	LK	LHLK (brain)	To central body	(Jeong et al. 2021)
	MIP	Brain neurons	Ecdysone‐dependent	(Yip et al. 2022)
	Marmite	Brain neurons	?	(Francisco et al. 2022)
	NPF	Gut EECs	To IPCs via JH production	(J. Chen et al. 2024)
			To IPCs and AKH cells. Glucagon and lipid metabolism	(Yoshinari et al. 2021)
	PDF	VNC neurons	Via CC cells (APCs)	(Braco et al. 2022)
	sNPF	CN neurons	Acts on IPCs and APCs	(Oh et al. 2019)
Osmoregulation	CAPA	VNC neurosecr.	Renal tubules (PC)	(Kean et al. 2002)
	DH31	Gut EECs	Renal tubules (PC)	(Coast et al. 2001)
	DH44	VNC neurosecr.	Renal tubules (PC)	(Cabrero et al. 2002; Zandawala et al. 2018)
	GPB5	VNC neurosecr.	Hindgut	(Rocco and Paluzzi 2016; Sellami et al. 2011)
	ITP	LNCs, VNC efferents?	Renal tubules (SC and PC), hindgut?	(Gera et al. 2025)
	LK	VNC neurosecr.	Renal tubules (SC)	(Radford et al. 2002; Zandawala et al. 2018)
	NPF	Gut EECs?	Renal tubules	(Chintapalli et al. 2012)
	PDF	VNC efferents	Ureter muscle	(Talsma et al. 2012)
	RYa	VNC efferents	Hindgut?	(Veenstra and Khammassi 2017)
	TK	Gut EECs	Renal tubules (SC)	(Agard et al. 2024)
Gut function	DH31	Gut EECs	Defecation	(Kotronarou et al. 2023)
			Microbe sequestering	(Tleiss et al. 2024)
			Gut motility (during infection)	(Benguettat et al. 2018)
	DH44	MNCs	Defecation	(Dus et al. 2015)
	ITP	Brain LNCs, VNC efferents?	Defecation	(Galikova et al. 2018; Gera et al. 2025)
	PDF	VNC efferents	Defecation	(W. Zhang et al. 2014)
	TK	Gut EECs	Lipid metabolism	(W. Song et al. 2014)
			Immune response	(Kamareddine et al. 2018; Takada et al. 2024)
Stress	AKH	CC (APCs)	Via central complex (FB)	(Nelson et al. 2021)
	AstA	SEZ neurons	Via blood–brain barrier	(Alia et al. 2026)
	CAPA	VNC NSCs	Renal tubules: cold and osmotic stress	(Terhzaz et al. 2015)
	Corazonin	LNCs	Fat body: metabolic stress	(Kubrak et al. 2016; Zandawala et al. 2021)
	DH31	Gut EECs	Microbial stress	(Benguettat et al. 2018)
	DH44	MNCs and VNC neurons	Renal tubules: osmotic and metabolic stress	(Cannell et al. 2016; Zandawala et al. 2018)
	DILPs	Brain IPCs	Via fat body	Reviewed in (Grönke et al. 2010; Nässel and Vanden Broeck 2016)
	FMRFa	Mutant analysis	Heat stress sleep	(Lenz et al. 2015)
	ITP	Brain LNCs	Osmotic and metabolic stress	(Gera et al. 2025)
	LK	VNC neurons	Renal tubules: osmotic metabolic stress	(Cannell et al. 2016; Zandawala et al. 2018)
	NPF	Brain neurons	Autophagy, via DA neurons	(T. Chen et al. 2023)
Cardiac function	AKH	CC (APCs)	High fat‐induced arrhythmia	(Y. Zhao et al. 2023)
	CCAP	Peripheral neurons	Increase heartbeat	(Dulcis et al. 2005; H. Li et al. 2025)
	CRZ	LNC	Decrease heartbeat	(H. Li et al. 2025)
Nutrient sensing	AKH	Corpora cardiaca	Glucose	(S. K. Kim and Rulifson 2004)
	AstC	INSO neurons	Sodium sensing (gut)	(B. Kim et al. 2024)
		Gut EECs	Low nutrients	(Kubrak et al. 2022)
	Burs	Gut EECs	Carbohydrate	(Scopelliti et al. 2014a)
	CCHa2	Gut EECs	Carbohydrate, yeast	(Sano et al. 2015)
	CNMa	Gut enterocytes	Amino acids	(B. Kim et al. 2021)
	Corazonin	Brain LNCs	Fructose	(Miyamoto et al. 2012)
			Glucose	(Oh et al. 2019)
	DH31	Gut EECs	Amino acids	(H. ‐H. Lin et al. 2022)
			Reactive oxygen species	(Benguettat et al. 2018)
	DH44	MNCs	Food consumption	(Oh et al. 2021; Z. Yang et al. 2018)
	NPF	Gut EECs	Carbohydrate	(Yoshinari et al. 2021)
	sNPF	CN neurons	Glucose	(Oh et al. 2019)
Diapause	AKH	Corpora cardiaca	Fat body?	(Kučerová et al. 2016)
	AstC	Clock neurons	Temperature sensing	(Meiselman, Alpert, et al. 2022)
	DH31	LNCs	Via JH in CA	(Kurogi et al. 2023)
	DILPs	IPCs	Via JH in CA	(Kubrak et al. 2014; Kurogi et al. 2021; Nagy et al. 2019)
	PDF	Clock neurons	Via IPCs	(Nagy et al. 2019; Ojima et al. 2018)
	sNPF	Clock neurons	Via IPCs	(Kurogi et al. 2021; Nagy et al. 2019)
Ovary regulation	CNMa	Fat body	Ovulation	(Grmai et al. 2024)
	DILPs^a^	IPCs	CA via JH	(Grönke et al. 2010)
	NPF	Gut EECs	Egg production	(Hoshino et al. 2023)

*Note:* This table is updated and altered from (Nässel, Pauls, and Huetteroth 2019) and Nässel and Zandawala (2019). Peptide abbreviations are in the list of acronyms.

Other acronyms: APCs, AKH‐producing cells; CA, corpora allata; DA, dopamine; FB, fan‐shaped body; GR, Gr5a, Gr64f and Gr66a, gustatory receptors; ILPs, insulin‐like peptides; IPCs, insulin‐producing cells; JanuAstA, AstA‐expressing interneurons; JH, juvenile hormone; LN, local neurons; LNCs, lateral neurosecretory cells; MB, mushroom bodies; MNCs, median neurosecretory cells; MP1/MP3, DSK‐expressing interneurons, OSN, olfactory sensory neurons; PC, principal cells; PN, projection neurons; SC, stellate cells; SEZ, subesophageal zone; SMP‐TK, TAG, thoracico‐abdominal ganglion; TK‐expressing interneurons; VM2, pheromone‐sensitive glomerulus in antennal lobe.

^a^ The literature on insulin signaling is extensive; only a few reviews are given here.

**TABLE 5:** Roles of NPHs in larval brain neurosecretory cells (NSC).

**NPH**	**Cell** ^a^	**Role in larva**	**Reference**
DH44	MNC	Glucose sensing	(Poe et al. 2024)
		Light avoidance (nutrient‐dependent)	(Imambocus et al. 2025)
		Arousal/activity (via clock input)	(Poe et al. 2023; Poe et al. 2024)
ILP2, 3, 5	MNC	Development, nutrient‐dependent growth, metabolism	(Brogiolo et al. 2001; Dunham and Bland 2026; Géminard et al. 2006; Grönke et al. 2010; Koyama and Mirth 2018; Tennessen and Thummel 2011)
EH^b^	Medial ventral	Ecdysis cascade	(McNabb et al. 1997; Scott et al. 2020)
CRZ	Lateral (LNC)	Fructose sensing	(Kulshrestha et al. 2024; Mishra et al. 2013)
		Growth via PTTH and ecdysone	(Imura et al. 2020)
		Growth (mechanism?)	(Megha et al. 2019)
ITP	LNC	Antidiuretic (hindgut)	(Okamoto et al. 2026)
DH31^C^	LNC	Immune response (IMD pathway) gut EECs	(Tleiss et al. 2024)
PTTH^b^	LNC	Ecdysis/developmental timing	(McBrayer et al. 2007; Yamanaka, Rewitz, et al. 2013)
		Phototaxis (light avoidance)	(Yamanaka, Romero, et al. 2013)
CAPA	Subesophageal	Calcium mobilization	(Okamoto et al. 2026)
Hugin‐PK^d^	Subesophageal	Ecdysis	(Meng et al. 2002)
		Food avoidance	(Surendran et al. 2017)
		Food search/feeding	(Hückesfeld et al. 2016; Schlegel et al. 2016; Schoofs et al. 2014)
		Locomotion	(Schoofs et al. 2014)
AKH	Corpora cardiaca	Glucose sensing	(J. Kim and Neufeld 2015; S. K. Kim and Rulifson 2004)
		Carbohydrate metabolism	(J. Kim and Neufeld 2015; S. K. Kim and Rulifson 2004)
		ILP3 release	(S. K. Kim and Rulifson 2004)
		UV stress (nociception)	(Guntur et al. 2015)
		Locomotion (infection‐induced)	(Ibrahim et al. 2018)

*Note:* Peptide acronyms in list of abbreviations. Nt, not tested.

^a^ Functional role mediated via the NSCs listed unless other cells indicated.

^b^ These NSCs are present only in larvae and pharate adults.

^c^ Important, not NSCs (only EECs tested).

^d^ Not clear which of the Hugin‐PK neurons in SEZ cluster mediate the response.

**TABLE 6:** Roles of NPHs in adult brain NSCs.

NPH	Cell^a^	Roles in adult	Reference
DMS	Medial (MNC)	Feeding (female post‐mating; crop)	(Hadjieconomou et al. 2020)
DH44	MNC	Food search/feeding	(Dhakal et al. 2022; Dus et al. 2015; Oh et al. 2021; Z. Yang et al. 2018)
		Sugar sensing	(Dus et al. 2015; Oh et al. 2021)
		Amino acid sensing	(Z. Yang et al. 2018)
		Defecation	(Dus et al. 2015)
		Lipid storage	(Nath et al. 2025)
		Rest‐activity rhythm (downstream clock)	(Barber et al. 2021; Cavanaugh et al. 2014; King et al. 2017)
		Sperm storage (females)	(K. ‐M. Lee et al. 2015)
DSK	MNC	Satiety induction	(Söderberg et al. 2012)
		Aggression	(P. Agrawal et al. 2020; J. Luo et al. 2014)
ILP1^b^	MNC	Metabolism, lifespan	(Liao et al. 2020; Y. Liu, Liao, et al. 2016; Post et al. 2019)
ILP2, 3, 5^c^	MNC	Food search/feeding	(González Segarra et al. 2023; X. Li et al. 2024; Nässel and Vanden Broeck 2016; Owusu‐Ansah and Perrimon 2014)
		Thirst/drinking	(González Segarra et al. 2023)
		Metabolism	(Géminard et al. 2006; Nässel and Vanden Broeck 2016; Owusu‐Ansah and Perrimon 2014; Teleman 2010)
		Ethanol intoxication	(Corl et al. 2005)
		Courtship/reproduction	(S. ‐L. Chen et al. 2022; Grönke et al. 2010; Lebreton et al. 2015; L. Zhang et al. 2022)
		Activity/sleep	(Barber et al. 2021; Biglari et al. 2025; Yamaguchi et al. 2022)
		Learning	(Chambers et al. 2015; Naganos et al. 2012; Tanabe et al. 2017)
		Stress	(Grönke et al. 2010; Nässel and Vanden Broeck 2016)
		Reproductive diapause	(Kubrak et al. 2014; Kurogi et al. 2021; Nagy et al. 2019)
		Aging/lifespan	(Owusu‐Ansah and Perrimon 2014; Partridge et al. 2011; Tatar et al. 2003; Tatar et al. 2001)
EH	Medial ventral	Not in adults	—
CRZ	Lateral (LNC)	Feeding	(Kubrak et al. 2016)
		Glucose sensing, act by colocalized sNPF	(Oh et al. 2019)
		Activity/sleep	(Afonso et al. 2015)
		Learning (mating reward, males)	(Zer‐Krispil et al. 2018a)
		Ethanol‐related behavior	(McClure and Heberlein 2013; Sha et al. 2014)
		Courtship	(Bergland et al. 2012; Bonheur et al. 2023; H. ‐H. Lin et al. 2022; T. Zhang et al. 2024)
		Colocalized sNPF (not CRZ): Regulation of AKH and ILP secretion; thereby glucose homeostasis	(Kapan et al. 2012; Oh et al. 2019)
		Metabolism	(Kubrak et al. 2016; Zandawala et al. 2021)
		Metabolic stress	(Kubrak et al. 2016; Zandawala et al. 2021)
		Osmotic and ionic homeostasis (via CAPA neurons in VNC)	(Zandawala et al. 2021)
		Nutrient sensing (fructose)	(Miyamoto et al. 2012)
ITP	LNC	Thirst/drinking, feeding, osmoregulation (antidiuretic), metabolism, stress, defecation	(Galikova et al. 2018; Gáliková and Klepsatel 2022; Gera et al. 2025)
DH31	LNC	Diapause (via juvenile hormone)	(Kurogi et al. 2021)
PTTH	LNC	Not in adults	—
Hugin‐PK^d^	Subesophageal	Locomotor activity (downstream clock and DH44)	(King et al. 2017)
		Sleep (downstream sleep homeostat)	(Schwarz et al. 2021)
AKH	Corpora cardiaca	Taste (Gr5a)	(Bharucha et al. 2008; Jourjine et al. 2016; Yu et al. 2016)
		Food search/feeding	(Bharucha et al. 2008; Gáliková et al. 2015; G. Lee and Park 2004; Pauls et al. 2021; Yu et al. 2016)
		Activity/sleep (via OA)	(Pauls et al. 2021)
		Locomotion (nutrient‐dependent)	(Isabel et al. 2005; G. Lee and Park 2004)
		Learning (via OA)	(Meschi et al. 2024)
		Metabolism	(Braco et al. 2022; Gáliková et al. 2015; Nelson et al. 2021; Wat et al. 2021)
		Stress	(Nelson et al. 2021)
		Heart function	(Y. Zhao et al. 2023)
		Diapause	(Kučerová et al. 2016)

*Note:* Peptide acronyms in list of abbreviations.

^a^ Function mediated via the NSCs listed unless other cells indicated.

^b^ ILP1 is expressed in insulin‐producing cells (IPCs) during pupal stage and first week of adult life.

^c^ Not specified here which of the three DILPs co‐localized in the IPCs that mediate the response (the original papers in some cases specify).

^d^ Not clear whether action is due to NSCs or Hugin‐PK‐expressing interneuron.

The larval roles of NPHs produced by brain NSCs are primarily related to developmental timing, growth and ecdysis/molting, feeding (including nutrient sensing), metabolism, stress/nociception, immune responses, phototaxis, and locomotor activity (including clock‐dependent arousal and nociception‐induced escape). References to these functions are given in Table 5. Not surprisingly, the spectrum is much wider in adults, and the overlapping actions of specific NSCs (and their NPHs) are that both in larvae and adults, the MNCs‐expressing DH44 are involved in nutrient sensing and arousal (Cavanaugh et al. 2014; Dus et al. 2015; Oh et al. 2021; Poe et al. 2023; Poe et al. 2024), and LNCs‐producing CRZ and sNPF are sugar sensing (Kulshrestha et al. 2024; Mishra et al. 2013; Oh et al. 2019). The LNCs‐producing ITP act on the hindgut in larvae and renal tubules in adults to regulate water reabsorption (Gera et al. 2025; Okamoto et al. 2026). The cluster of Hugin‐pyrokinin (Hugin‐PK)‐producing cells of the subesophageal zone (SEZ) that includes NSCs regulates food search/feeding and locomotion in both stages, but it is not clear which specific cell type(s) (King et al. 2017; Martelli et al. 2017; Schoofs et al. 2014; see Tables 5 and 6). The IPC‐derived ILPs regulate aspects of metabolism and energy mobilization (and indirectly feeding) in both stages, but in the larva, primary roles are in nutrient‐dependent development and growth (summarized in Dunham and Bland 2026; Garelli et al. 2026; Grönke et al. 2010; Okamoto and Mizoguchi 2026; Owusu‐Ansah and Perrimon 2014), and in adults, many more roles have been discovered in behavior and physiology (see references in Table 6).

There are several NPH functions associated with NSCs that have been detected only in larvae, in addition to the ones of the PTTH and EH neurons. For example, CRZ regulates growth via PTTH neurons and ecdysone (Imura et al. 2020). DH44 is involved in a larval light avoidance mechanism (Imambocus et al. 2025). Finally, *Hugin* overexpression was found to disrupt ecdysis (Meng et al. 2002).

The repertoire of roles of NSC‐associated NPHs in adults has expanded into regulation of the more complex adult behavior and physiology, including sexual maturation and aging. For instance, additional to functions shown in the larva, the DH44‐producing MNCs are amino acid sensing and are involved in food search and feeding, regulation of lipid storage, defecation, and female sperm storage (see Table 6 for references). In adults, ILPs and the IPCs have mostly switched gears during metamorphosis and now regulate food search (including modulation of food odor sensitivity) and feeding (hunger/thirst), metabolism, reproduction (maturation and physiology but also courtship), activity/sleep, learning, stress responses, reproductive diapause, and aspects of aging (see Table 6 for references). Thus, the multifunctional ILPs are recruited for regulation of adult‐specific behavior and physiology, although they are released from the same set of NSCs as in the larva. Of note is the production of IPC‐derived ILP1 during the pupal and early adult stage (and in diapause) but no expression in larvae (Y. Liu, Liao, et al. 2016; Post et al. 2019).

There is an increase in the number and types of regulatory inputs (mediating internal states and external stimuli) to the IPCs during metamorphosis, leading to increased complexity in IPC (and ILP) signaling. Synaptic inputs to and/or regulators of *Drosophila* IPCs and other NSCs have been charted both in larvae and adults (Gera et al. 2025; Held et al. 2025; Nässel and Vanden Broeck 2016; Schlegel et al. 2016). A similar increase in regulatory inputs is likely to be seen also for the other adult NSCs.

Another example of changing functions has been found in the CRZ neurons, which in larvae regulate growth via PTTH‐producing neurons and ecdysone (Imura et al. 2020) and serve in sugar sensing (Kulshrestha et al. 2024; Mishra et al. 2013). In adults, they also sense sugar (Miyamoto et al. 2012) but in addition have multiple other functions, including regulation of food seeking and feeding, metabolism, activity/sleep, learning, stress responses, courtship, and ethanol‐associated behavior (Bonheur et al. 2023; Kubrak et al. 2016; H. ‐H. Lin et al. 2022; McClure and Heberlein 2013; Sha et al. 2014; T. Zhang et al. 2024).

Some of the brain NSCs co‐express further NPHs in adults (see Figure 2F). The IPCs produce ILP1, 2, 3, and 5 (Brogiolo et al. 2001; Y. Liu, Liao, et al. 2016), and a subpopulation of these also sulfakinin (DSK; Söderberg et al. 2012). The CRZ‐producing LNCs (l‐NSC^CRZ^) coexpress sNPF (Kapan et al. 2012; McKim et al. 2024); the LNCs‐producing ITP (l‐NSC^ITP^, also known as ALK) also express sNPF, TK, and LK (de Haro et al. 2010; Gera et al. 2025; Kahsai, Kapan, et al. 2010; McKim et al. 2024); and finally the Hugin cells of the SEZ (SEZ‐NSC^Hugin^) also produce sNPF (McKim et al. 2024). The roles of these additional NPHs have been studied in some cases. DSK in IPCs acts to induce satiety (Söderberg et al. 2012) and affects aggressive behavior (P. Agrawal et al. 2020; J. Luo et al. 2014). Furthermore, LK and TK in the l‐NSC^ITP^ cells modulate responses to metabolic and ionic stress (Kahsai, Kapan, et al. 2010), and ILP1 in IPCs displays functions specific to non‐feeding and non‐reproductive stages (Liao et al. 2020; Y. Liu, Liao, et al. 2016; Post et al. 2019).

Outside the brain, the endocrine cells of the corpora cardiaca produce AKH. AKH displays some conserved functions in the two stages (all are nutrient‐dependent), like regulation of glucose and lipid metabolism (Gáliková et al. 2015; J. Kim and Neufeld 2015; S. K. Kim and Rulifson 2004; G. Lee and Park 2004) and locomotion/foraging (nutrient‐ or stress‐dependent; Isabel et al. 2005; G. Lee and Park 2004; Nelson et al. 2021). However, additional functions have been explored in adults as seen in Table 6. These are regulation of taste, food search/feeding, activity/sleep, learning, stress, heart function, and diapause (references in Table 6). The endocrine cells of the corpora cardiaca also produce another peptide, limostatin, that suppresses ILP secretion from IPCs during nutrient restriction (Alfa et al. 2015).

Finally, it shall be emphasized that the NPHs that are released from brain NSCs are additionally produced by other types of neurons and gut EECs and therefore are involved in multiple functions (Figure 3), some of which, in a few cases, are similar in larvae and adults. Thus, for instance, ITP can be found in both NSCs and two types of clock neurons (Figure 3A–C), and DH31 can be found both in interneurons, NSCs of the VNC, and EECs of the gut (Figure 3C).

**FIGURE 3:**
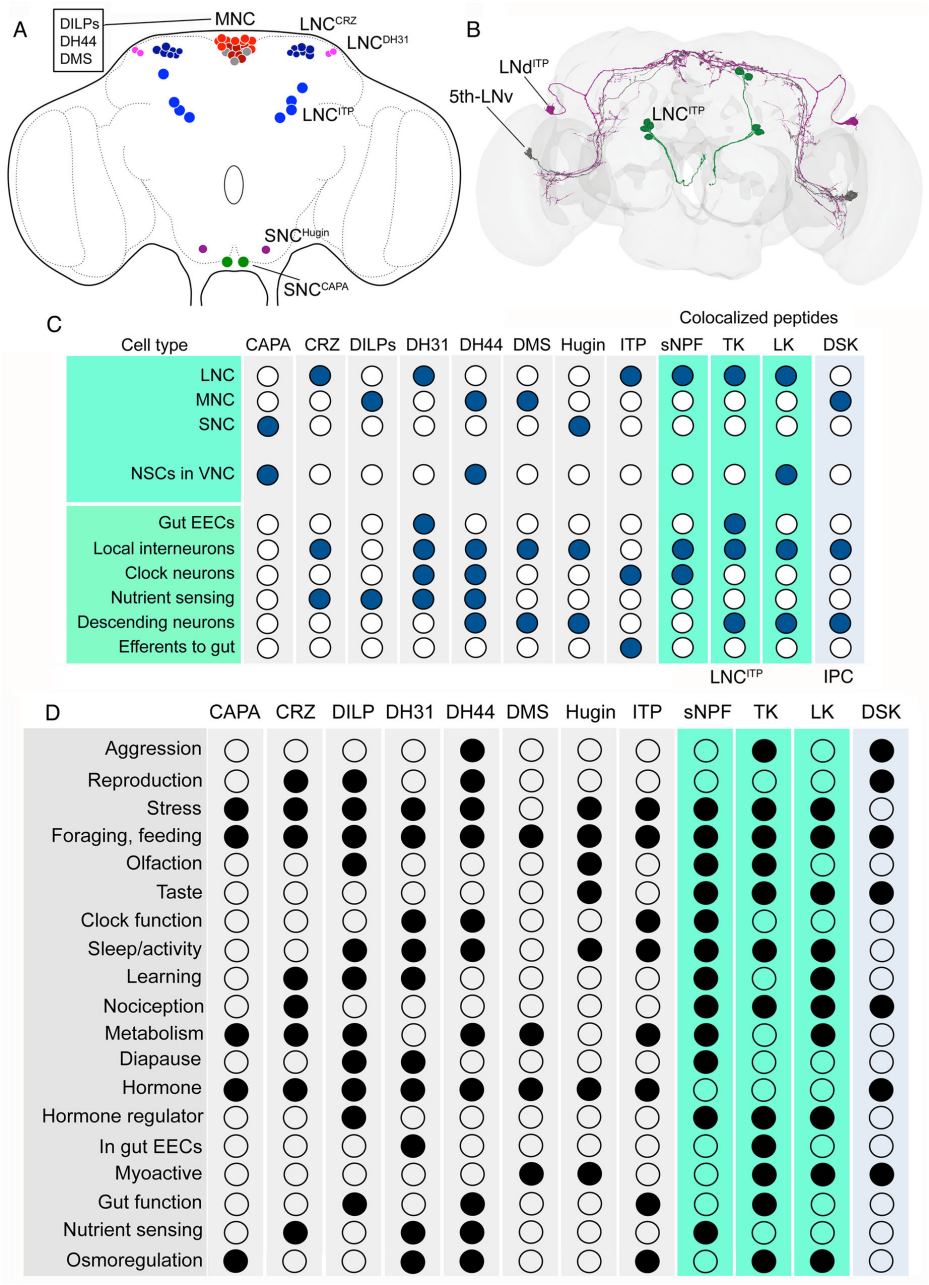
In adult flies, NPHs of NSCs are produced by additional cell types and also display multiple non‐hormonal functions. (A) The NSCs of the brain. Note that each of the LNC^CRZ^ clusters shown here includes three NSCs and four interneurons. (B) Ion transport peptide is produced in LNCs (LNC^ITP^) but also in clock neurons (5th‐LNv and LNd^ITP^). (C) NPHs of NSCs are expressed by multiple cell/neuron types. Two cell types (LNC^ITP^ and IPC) express additional peptides: sNPF, TK, LK, and DSK. Nutrient sensing refers to nutrient‐sensing cells. (D) NPHs of NSCs have multiple additional roles, many of which are non‐hormonal and based on release from different cell/neuron types (the colocalized peptides as in B). For references, see Table 6. This figure is compiled from Nässel (2025). (B is originally from Gera et al. 2025, with permission.)

## 5 NSCs in the VNC of Larvae and Adults and Their Functions

### 5.1 Numbers of Neurons, Their NPHs, and Distribution

In *Drosophila* and other insects, there are also peptidergic NSCs and other endocrine cells outside the brain and corpora cardiaca. Here, I will deal with NSCs in the VNC but ignore endocrine cells located outside the CNS, such as Inka cells in the trachea (Zitnan et al. 1996, 2003) and others located in thoracic nerve roots expressing, for example, ITP and AstA (Dircksen et al. 2008; Yoon and Stay 1995).

Three major types of NSCs can be distinguished in the VNC of insects (Nässel 1996) as shown for *Drosophila* in Figure 4: NSCs with axons that exit the VNC dorsally in a median tract (thus termed median NSCs) and NSCs with axons emerging via lateral nerves (lateral NSCs) and finally median posterior efferent neurons (not all diagnosed as NSCs). Such median and lateral NSCs in abdominal ganglia were described early on in the moth *Manduca sexta* (N. T. Davis et al. 1993; Figure 4B) and other insects (see Nässel 1996). The NPHs produced by these VNC neurons in *Drosophila* and *M. sexta* are listed in Figure 4B,C.

**FIGURE 4:**
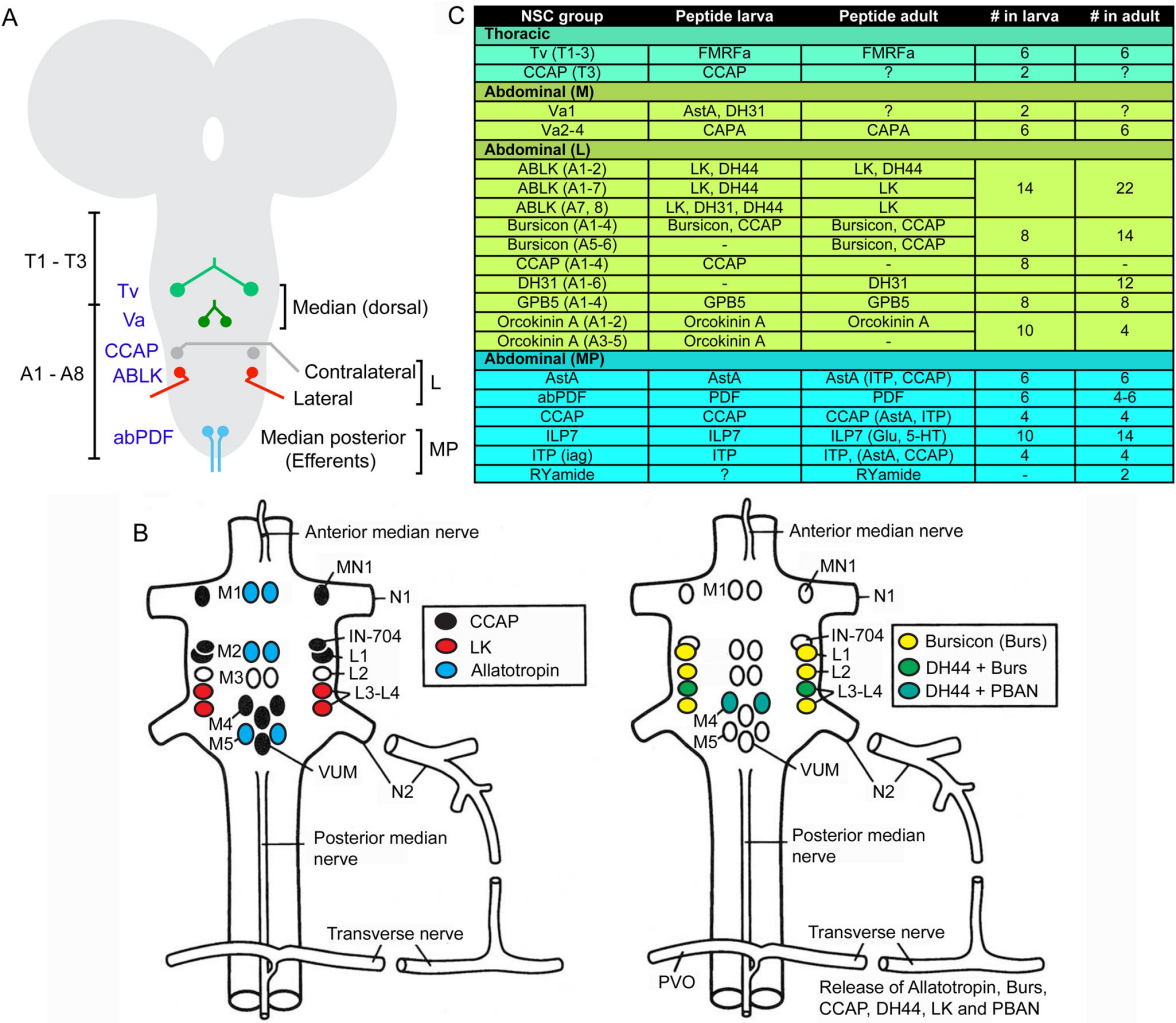
Classification of NSCs and efferent neurons in ventral nerve cord (VNC). (A) The major classes of NSCs and efferent neurons in thoracic (T1–T3) and abdominal (A1–A8) neuromeres, shown in the larval CNS. Note that in adults, the locations of the abdominal NSC cell bodies are not as distinct, and segmental and median‐lateral positions are hard to discern. Median NSCs (e.g., Tv and Va) have axons projecting dorsally (and medially) into perisympathetic organs (Tv) or transverse nerves (Va) in larvae and neurohaemal areas in adults. Lateral (L) NSCs (e.g., CCAP and ABLK) have axons that exit via lateral segmental nerves in larvae and fused lateral abdominal nerves in adults (note that axons of CCAP neurons exit on the contralateral side). Median posterior (MP) efferent neurons (e.g., abPDF) exit posteriorly toward the hindgut and/or reproductive tract via median abdominal nerves. Note that of these, an NSC role has only been shown for abPDF and possibly some of the ILP7 MP neurons (see text). Some of these efferents (listed in B) may directly act on the tissues that they innervate. (B) Peptidergic NSCs in an unfused abdominal ganglion of the moth *Manduca sexta* display a clear medial‐lateral organization. To support the division of *Drosophila* NSCs into lateral and median, an *M. sexta* abdominal ganglion is shown. These neurons are individually identifiable and have been assigned acronyms, L1–L4 for lateral and M1–M5 for median. Shown additionally are peptidergic motoneurons (MN1) and interneurons (IN‐704) as well as ventral unpaired median (VUM) neurons. Note that some of the NPHs shown here are colocalized in certain NSCs but shown here separately (e.g., in L3, L4 cells and others). The median NSCs send axons via the anterior median nerve to an anteriorly located transverse nerve perivisceral organ (PVO) and the lateral ones via nerve 2 (N2) to the posterior transverse nerve (and some additional release sites). The NPHs allatotropin and pheromone biosynthesis activating neuropeptide (PBAN) are not found in *Drosophila* (although PBAN is a pyrokinin‐like peptide). This figure is slightly altered from Nässel (1996), which in turn was based on data from N. T. Davis et al. (1993), Taghert and Truman (1982), and Veenstra et al. (1994). (C) Table listing the peptidergic NSCs and efferents of the VNC in *Drosophila* larvae and adults. There are thoracic and abdominal lateral (L) and median (M) neurons. In brackets (abdominal lateral), the neuromere distribution of cells is provided. Note that only a few of the NSC groups have been given names (Tv, Va, ABLK, abPDF, iag); the rest are listed after the primary peptide they produce (colocalized peptides were commonly discovered later). Note that in some cases, the peptide was not found (‐), in other cases not investigated (?). For peptide acronyms, see list. For literature references, see main text (see also Figure 5).

Here, we shall first look at the median NSCs located in the thoracic and abdominal neuromeres of the *Drosophila* VNC that have dorsal/median axon terminations in bona fide neurohemal organs (perisympathetic organs, PSOs) or peripheral axon termination areas (neurohemal areas; see Figures 4, 5, 6). Next, I describe NSCs with axons emerging via lateral nerves of the VNC and with terminations on tissues outside the CNS, for instance, on muscles and body wall (not in dedicated PSOs; Figure 5). These thoracic and abdominal neurons are collectively referred to as NSCs although hormonal functions of their NPHs have not been demonstrated for all neurons listed here. In Section 6, I describe the third type, the efferent VNC neurons with cell bodies located posteriorly in the abdominal neuromeres and axons that target the intestine or reproductive organs (Figure 7).

**FIGURE 5:**
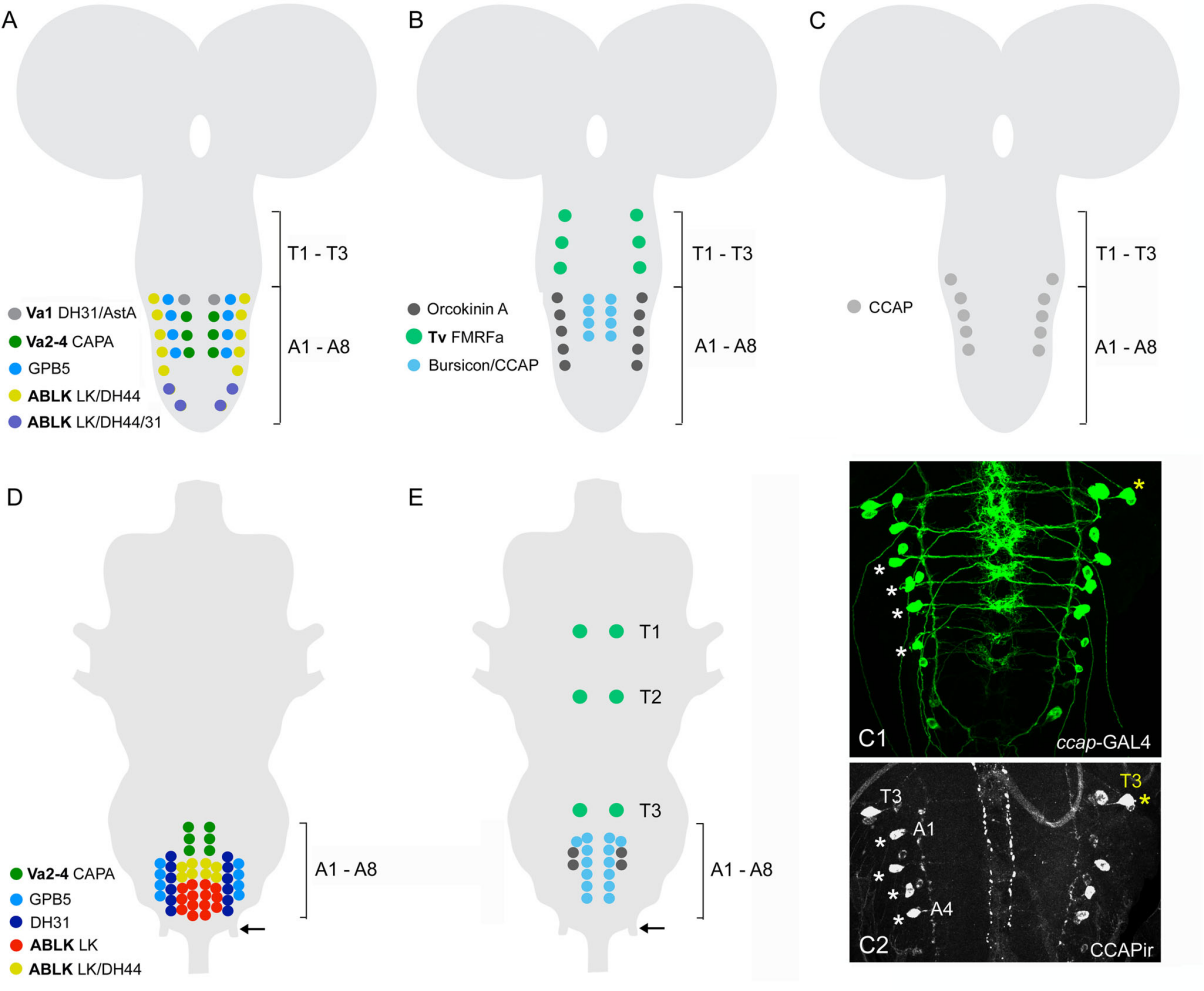
Peptidergic NSCs in the larval and adult ventral nerve cord. (A–C) Larval NSCs. In thoracic neuromeres (T1–T3), only three pairs of FMRFamide‐producing NSCs have been identified (Tv neurons) and one pair of CCAP‐producing neurons. In abdominal neuromeres (A1–A8), NSCs are located segmentally laterally and more medially. Some of the NSCs have been assigned names (shown in bold font). The peptide acronyms are: diuretic hormone 31 (DH31) and 44 (DH44), capability (CAPA), glycoprotein hormone B (GBP5), leucokinin (LK), crustacean cardioactive peptide (CCAP). Note that the Va and Tv neurons send axons to the dorsal midline where they terminate in perisympathetic organs (Tv neurons) and dorsal transverse nerves (Va neurons); see Figure 6. The lateral NSCs send axons through segmental nerves to body wall muscle. Note that the anterior ABLKs coexpress LK and DH44 (and octopamine), and the ones in A6 and A7 additionally express DH31 (Tsukasa et al. 2026; Zandawala et al. 2018). Note that it is not clear whether the abdominal CCAP neurons in C are the same as the bursicon/CCAP neurons in B. C1 and C2: Confocal images of CCAP‐expressing neurons in the VNC of the larva displayed with *ccap*‐GAL4 (C1) and CCAP‐immunolabeling (C2) in the same specimen. The asterisks indicate the CCAP neurons in T3 and A1–A4 in 4C, which display stronger immunolabeling. Note that a pair of neurons in T3 (yellow asterisk) also has axons to the periphery like the ones in A1–4 (see Karsai et al. 2013; Vömel and Wegener 2007). (D and E) Adult NSCs. The same neuropeptides are produced in adults, but the numbers of NSCs differ in some cases from the larva (ABLKs, bursicon/CCAP and Orcokinin A neurons; Chen et al. 2015; de Haro et al. 2010; Luan et al. 2006; Sellami et al. 2011; Zandawala et al. 2018). Also, the co‐expression of peptides differs for the ABLK neurons, and the NPH of anterior Va1 neurons has not been determined in adults. Note that in adults, the Va and Tv neurons supply axon terminations to extensive neurohemal areas in the dorsal neural sheath of the ventral nerve cord (see Figure 6). The remaining neurons in A1–A8 send their axons via the lateral abdominal nerves (arrows) to peripheral targets.

**FIGURE 6:**
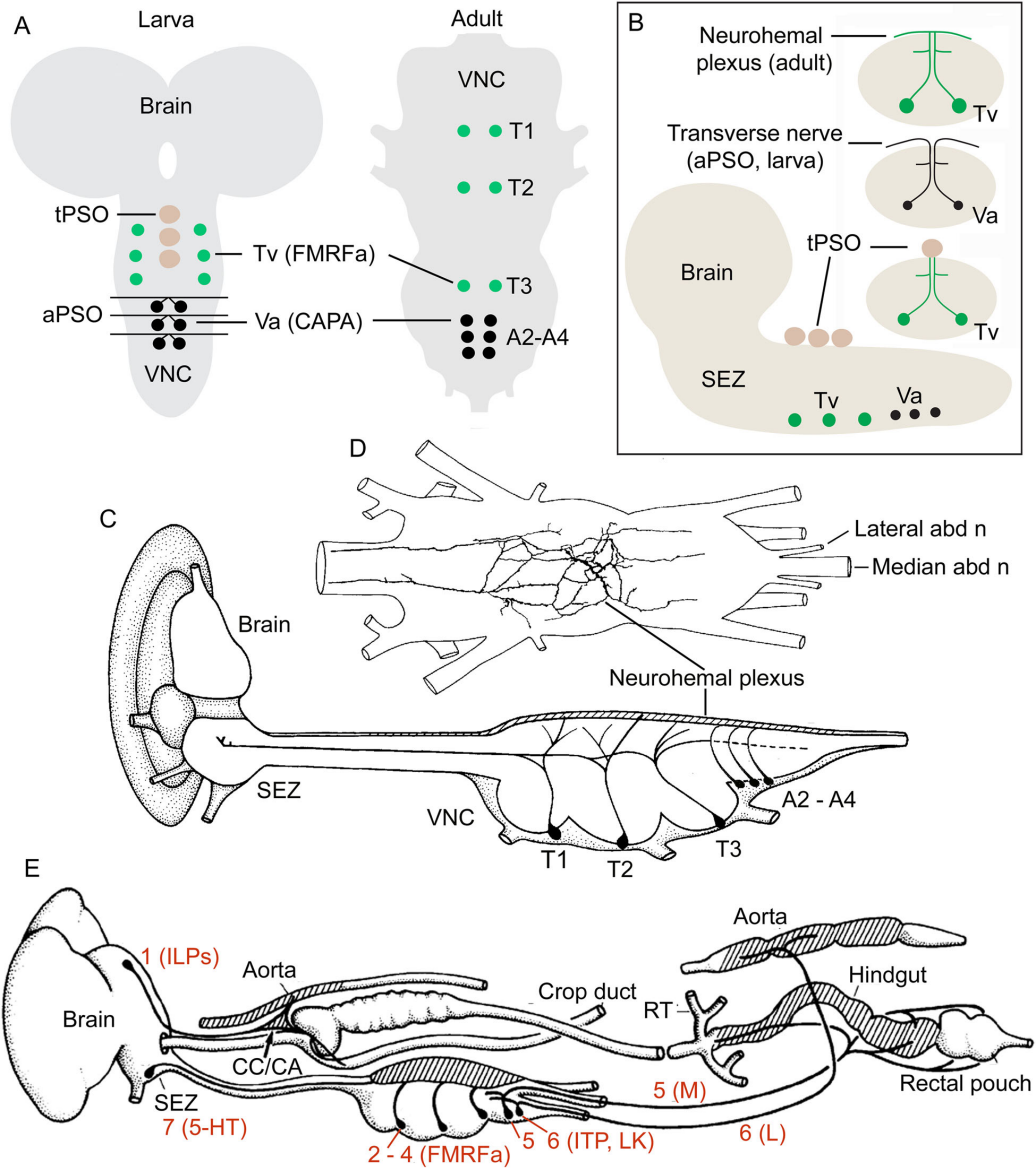
Release sites of larval and adult NSCs of the ventral nerve cord (VNC) change during metamorphosis. (A and B) Larval and adult Tv and Va neurons express FMRFamide and capability (CAPA), respectively (shown in dorsal view). In the larva, Tv neurons of thoracic neuromeres T1–T3 supply axon terminations to dorsal perisympathetic organs (tPSO; also known as neurohemal organs), whereas the adult Tv neurons supply axon terminations to a dorsal neurohemal plexus covering the VNC (see panels B–D). In larvae, the Va neurons of abdominal neuromeres A2–A4 send their axons dorsally into median transverse nerves (see also panel B). (B) Side view and transverse views of the VNC showing the tPSOs, transverse nerve, and dorsal neurohemal plexus. Note that in the side view, the abdominal transverse nerves are not shown. (C) Side view of the adult VNC with Tv neurons of thoracic neuromeres T1–T3 and Va neurons of A2–A4. Both neuron types send axons into the dorsal neural sheath where they arborize and form varicosities, as depicted in D (note that only the Tv neuron plexus is shown in D). (D) Dorsal view of the VNC with the thoracic neurohemal plexus. (E) Release sites of different types of NSCs (numbered 1–7). Release sites (axon terminations) are shown crosshatched in aorta, dorsal surface of VNC, and hindgut). The examples of NSCs shown here include a set of four serotonergic (5‐HT) neurons in the SEZ that send axons to the same neurohemal area as the FMRFamide‐producing Tv neurons. The posterior neurons (5, ITP and 6, LK) exit via median (M) or lateral (L) abdominal nerves, respectively, to innervate dorsal aorta (heart) and body wall muscles (L) and hindgut (M). Further acronyms: CC, corpora cardiaca; CA, corpora allata. RT, renal tubules (shown truncated). (A) and (B), original. (C) and (D) from Lundquist and Nässel (1990), (E) from Nässel (1996), all with permission.

**FIGURE 7:**
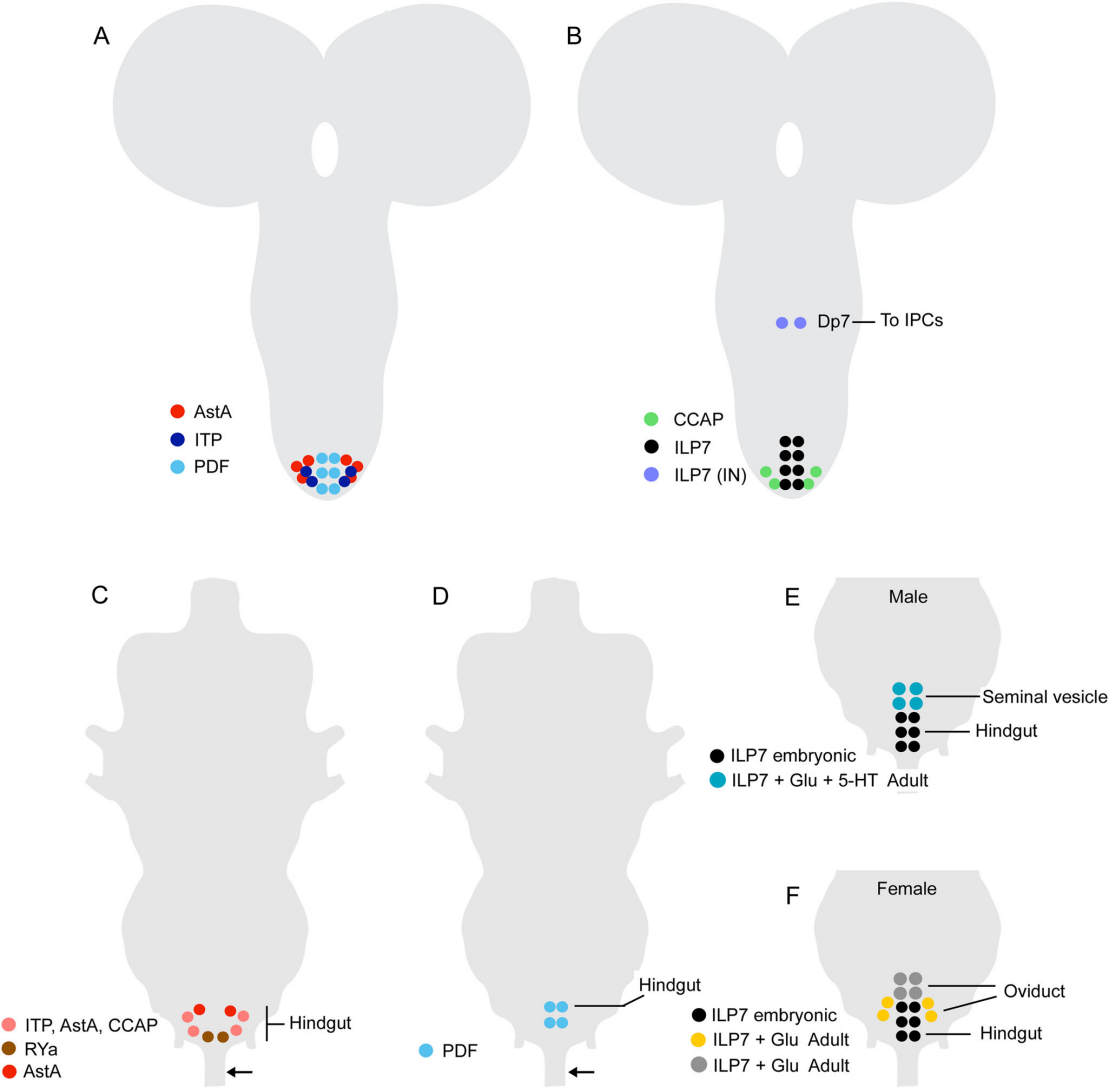
Posterior abdominal efferent neurons and NSCs that produce NPHs in larvae and adults. In both stages, sets of neurons in abdominal neuromeres send axons to the hindgut (and associated structures, e.g., rectal pads in adults) and in adults also to the reproductive tract (ILP7 neurons) via the median abdominal nerve(s). Some of these may serve as NSCs where the peptides have functions as circulating hormones (only shown for PDF and maybe ILP7), but the majority of the neurons seem to act locally on targeted tissues. (A and B). Larval neurons. Acronyms: AstA, allatostatin A; ITP, ion transport peptide; PDF, pigment‐dispersing factor; CCAP, crustacean cardioactive peptide; ILP7, insulin‐like peptide 7. Note that the two Dp7 neurons in B are not efferents but ascending interneurons that target the insulin‐producing cells (IPCs) in the brain (they are included for a function related to in the text). Note that a possible co‐expression of the peptides in A is possible, similar to adults, but needs to be confirmed. RYa, RYamide. (C and D) Adult efferents and NSCs that target hindgut and/or reproductive tissues. The PDF‐producing cells seem to release hormonal PDF that acts on Malpighian tubules (Talsma et al. 2012). The axons of these neurons exit via the median abdominal nerves (arrows). Acronyms as in (A and B). (E and F). A sex dimorphism has been demonstrated in the adult ILP7 neurons where embryo‐ and adult‐derived neurons exist. In males (E), the adult ILP7 neurons co‐express glutamate and serotonin (5‐HT) and innervate the seminal vesicle, the embryonic ones produce only ILP7 and innervate the hindgut. In females (F), two pairs of additional adult cells (yellow) co‐express ILP7 and glutamate, and they and another set of adult neurons innervate the reproductive tract (oviduct muscle), and the embryonic ones innervate the hindgut. Note that proctolin has also been detected in six efferent neurons in the VNC of larvae and adults (Anderson et al. 1988; Taylor et al. 2004) and innervate the hindgut musculature (Anderson et al. 1988) but are not shown here (maybe colocalization in the PDF neurons?).

In larvae, sets of segmental NSCs in the thoracic and abdominal neuromeres with dorso‐median‐projecting axons can release their peptide hormones via axon terminations in dorsal structures: spherical PSOs in thoracic neuromeres and dorsal bifurcating transverse nerves in the abdominal ones (Figure 6). In thoracic neuromeres (T1–3), only three pairs of peptidergic NSCs (Tv neurons) have been clearly identified so far, both in larvae and adults (but see below); these produce extended FMRFamides, also known as dFMRFa (Figures 5 and 6; Lundquist and Nässel 1990; Schneider et al. 1993; K. White et al. 1986). It can be mentioned that using an *itp*‐GAL4 driver (*ITP‐RC*), an additional set of Tv‐like neurons (dFMRFamide‐negative) was detected in T1–3 of adults (larvae not tested), but these did not label with ITP antiserum (Gera et al. 2025), so the NPH is still unknown. One pair of neurons expressing CCAP in T3 of larvae send axons contralaterally to peripheral targets (body wall muscles M12 and M13), but it is not clear whether these are NSCs (Karsai et al. 2013; Vömel and Wegener 2007). In abdominal neuromeres 1–4, four pairs on NSCs (Va neurons) with dorso‐median axons have been detected (Kean et al. 2002; Santos et al. 2006). The anterior‐most pair (Va1) produces diuretic hormone 31 (DH31) and allatostatin A (AstA; Gabilondo et al. 2018) and the three following (Va2‐4) produce *capability* gene products, the CAPA peptides 1 and 2 (also known as periviscerokinins 1 and 2; Capa‐PVK1 and 2) and pyrokinin 1 (PK‐1; Kean et al. 2002; Predel and Wegener 2006; Santos et al. 2006; Figure 5A).

In addition, there are peptide‐producing lateral NSCs in the larval abdominal neuromeres A1–A8 with axons exiting through segmental lateral abdominal nerves to terminate on body wall muscle (see Cantera and Nässel 1992; Elliott et al. 2021; Sellami et al. 2011). One set of lateral NSCs produces LK with colocalized DH44 and octopamine in seven pairs of so‐called ABLK neurons (de Haro et al. 2010; K. Li et al. 2023; Zandawala et al. 2018), and the two last pairs in A6 and A7 additionally express DH31 (Tsukasa et al. 2026; Figure 5A). The other lateral neurons produce glycoprotein A2 and B5 (GPA2 and GPB5; Díaz‐de‐la‐Peña et al. 2020; Sellami et al. 2011; Tsukasa et al. 2026), orcokinin A (J. Chen et al. 2015; Tsukasa et al. 2026), crustacean cardioactive peptide (CCAP; Karsai et al. 2013), and finally a set of neurons with colocalized bursicon and CCAP (Karsai et al. 2013; C. W. Luo et al. 2005; Moris‐Sanz et al. 2015; Figure 5B,C). An atlas of the distribution of NPHs in neuronal cell bodies of the larval brain and VNC of *Drosophila* is provided in D. Park et al. (2008).

In the adult VNC, similar sets of NSCs have been detected, with a few notable differences. The thoracic FMRFamide‐expressing Tv neurons and the abdominal CAPA‐expressing Va neurons in A2‐4 can also be found in adults, although the median nerve disappears during metamorphosis. Their axon terminations are remodeled and arborize extensively in the dorsal neural sheath, thereby forming neurohemal release areas, not PSOs (Figure 6C,D; Lundquist and Nässel 1990; Santos et al. 2006), also shown earlier in more detail in blowflies (Nässel et al. 1988). No NPH has been detected in the adult Va1 neurons, but the Va2‐4 still produce *Capa* products and have axon terminations branching in the dorsal neuronal sheath of the posterior VNC (Santos et al. 2006). The lateral NSCs of A1–A8 in adults send their axons through a pair of fused lateral posterior abdominal nerves (Figure 6E) and terminate on various body wall targets (in those cases, it has been determined). In adults, these sets of NSCs produce LK, DH44, DH31, GPB5, orcokinin A, bursicon, and CCAP (J. Chen et al. 2015; de Haro et al. 2010; Díaz‐de‐la‐Peña et al. 2020; Luan et al. 2006; Sellami et al. 2011; Zandawala et al. 2018), but the number of cells is different in some cases (Figure 5). The 14 ABLKs of the larva increase to 22 NSCs in adults (eight adult‐specific), and only a subset of the ABLKs colocalize LK and DH44 (Zandawala et al. 2018). The DH31 neurons appear to be novel in adults. Although two pairs of posterior ABLKs co‐express DH31 in larvae (Tsukasa et al. 2026), this has not been shown in adults. The bursicon/CCAP neurons increase from 8 to 14, and finally the orcokinin A neurons decrease from 10 to 4 (Figure 5; J. Chen et al. 2015). Further peptidergic neurons with efferent axons to abdominal targets located in the median posterior neuromeres (Figure 5) are discussed in Section 6.

### 5.2 Functions of Larval and Adult NSCs of the VNC

In larvae, NSCs of the VNC that produce bursicon, CCAP, LK, FMRFa, and GPA2/GPB5, shown in Figure 5, play roles in development, especially in regulation of molting (Diao et al. 2017; D. H. Kim et al. 2015; Y. ‐J. Kim et al. 2006; J. H. Park et al. 2003; Vandersmissen et al. 2014; see Table 2). Further details of the action of these peptides will be given in Section 7.1. Additionally, the ABLK neurons (with colocalized DH44) are regulators in larval nociception escape behavior and rolling (avoidance; Imambocus et al. 2025; Imambocus et al. 2022; K. Li et al. 2023) as will be detailed in the next section. Finally, the larval CAPA neurons regulate calcium mobilization in the Malpighian tubules (Okamoto et al. 2026).

Interestingly, the corresponding adult VNC neurons serve a wider array of functions that have not been demonstrated in larvae (possibly for lack of trying). LK and DH44 (released from ABLKs) and Capa‐PVK1 and 2 (from Va cells) are diuretic hormones acting on different cells of the renal (Malpighian) tubules (Cabrero et al. 2002; Kean et al. 2002; Radford et al. 2002; Zandawala et al. 2018) and GPB5 acts as an antidiuretic (water reabsorption) on the hindgut (Rocco and Paluzzi 2016). Capa‐PVKs, DH44, and LK from VNC neurons also regulate osmotic stress (Cannell et al. 2016; Terhzaz et al. 2015; Zandawala et al. 2018). Additional functions of NSCs of the adult VNC are: CAPA neurons regulate metabolism via corpora cardiaca and AKH signaling (Koyama et al. 2021), ABLKs are part of circuitry modulating a mechanosensory response during mating (C. Liu et al. 2020), DH44 regulates feeding (Zandawala et al. 2018), and FMRFamide from Tv neurons signals to fat body to regulate metabolism (T. Song et al. 2023). Thus, the only functions that are remotely related in larvae and adults are those of CAPA‐PVKs acting on Malpighian tubules (but in different ways) and LK partaking in mechanosensory processing (nociception and avoidance). Again, the lack of further overlapping functions may be due to a shortage of studies in larvae.

### 5.3 ABLKs: An Example of Specific Set of NSCs in the VNC That Display Different Functions in Larvae and Adults

Here, I present in more detail an example of a small set of NPH‐producing VNC neurons, the ABLKs, that change functional roles during the larval‐to‐adult transition. The ABLKs are a set of segmental LK neurons in the seven anterior abdominal neuromeres (Cantera and Nässel 1992; de Haro et al. 2010) that have received quite a bit of attention both in larvae and adults and also during developmental molts (see Section 7.1). In contrast, the two pairs of brain LK neurons (LHLK and SELK) and the four pairs of LNCs (designated ALKs) that express variable levels of LK have been primarily studied in adults (see Nässel 2021).

In larvae, there are seven pairs of ABLKs laterally in the neuromeres A1–A7, whereas in adults, there are four additional pairs in the anterior neuromeres, yielding a total of 22 neurons (Figure 5; de Haro et al. 2010; Zandawala et al. 2018). The adult ABLKs are of different morphological types as seen by stochastic labeling (J. Chen et al. 2025). One type connects to the brain, the other two are local to the VNC, but all seem to have efferent axons with peripheral targets. Thus, there might be functional subtypes of adult ABLKs, while in larvae this has not been explored.

In larvae, all the ABLKs co‐express LK and DH44 (Zandawala et al. 2018) and octopamine (indicated by expression of tyrosine decarboxylase 2; K. Li et al. 2023), and the two last pairs in A6 and A7 additionally express DH31 (Tsukasa et al. 2026), whereas in adults, only a portion of the anterior ABLKs express DH44 (Zandawala et al. 2018; Figure 5). This differential peptide/octopamine expression may further diversify the functions of the ABLKs (Figure 7B). The axons of the larval ABLKs terminate on the segmental body wall muscle 8 (Cantera and Nässel 1992; de Haro et al. 2010), whereas in adult *Drosophila*, it has not been determined in detail where they terminate. In adults of another dipteran fly, *Phormia terraenovae*, the ABLK axons terminate on the dorsal vessel (lateral heart nerves), around tracheal spiracles and in neurohemal regions of the abdominal body wall (Cantera and Nässel 1992), suggesting release of LK as a circulating hormone. Judging from the morphology, the larval ABLKs may act locally on muscle 8 in each abdominal segment 1–8 rather than be a substrate for hormonal peptide release into the circulation. In both stages, LK may additionally act locally in circuits of the CNS.

#### 5.3.1 Larval Functions of ABLKs

Analysis of LK function in larval *Drosophila* has implicated roles of ABLKs in escape locomotion, such as light avoidance (Imambocus et al. 2022, 2025), heat nociception (rolling behavior; K. Li et al. 2023), and ABLKs respond to optogenetic activation of nociceptive cells (Y. Hu et al. 2020). The response to short‐wavelength light is mediated to the ABLKs via a dorsal pair of abdominal ILP7 neurons (Dp7) that also express sNPF (Imambocus et al. 2022; see Figure 7B). The heat‐induced facilitation of nociceptive rolling behavior was shown to also depend on the colocalized DH44 and octopamine in the ABLKs (K. Li et al. 2023). Furthermore, the ABLKs modulate turning (in response to blue light) in crawling larvae (Okusawa et al. 2014). The ABLKs are activated by serotonergic neurons via the 5HT1B receptor to induce this turning (Okusawa et al. 2014), and GABAergic inhibition to gate sensory input is mediated by the ionotropic GABA receptor RDL (Y. Hu et al. 2020). It is not clear whether the ABLKs induce escape and turning by direct action on body wall muscle (as motoneurons) or via action in circuits within the VNC and subsequent activation of motoneurons. LK from ABLKs furthermore regulates different aspects of ecdysis and post‐ecdysis behavior during development (D. H. Kim et al. 2015, 2018; Y. ‐J. Kim et al. 2006) as will be discussed in Section 7.1. In summary, it appears as if larval ABLKs act as both interneurons and efferents that target specific muscles, but no hormonal actions have been proposed so far. Thus, it is possible the ABLKs acquire their neurosecretory phenotype during metamorphosis, but this requires future experimental verification.

#### 5.3.2 Adult Functions of ABLKs

In adults, LK and DH44 of the ABLKs regulate ion and water homeostasis via action on Malpighian tubules and play roles in osmotic stress (Cabrero et al. 2002; Cannell et al. 2016; Radford et al. 2002; Zandawala et al. 2018). Since the Malpighian tubules are not innervated, LK and DH44 can only act as hormones via the circulation. Thus, the adult ABLKs are bona fide NSCs. The ABLKs also mediate sensory inputs from uterine sensory neurons to interneurons that trigger a wing defense response in mated female flies (C. Liu et al. 2020). Finally, ecdysone‐regulated ABLKs act on female‐specific pC1 neurons in the brain to reduce mating receptivity in young sexually immature female flies (J. Chen et al. 2025). Both these LK actions in female reproductive behavior appear to be interneuronal, not hormonal. Thus, adult ABLKs play several different roles both as interneurons and NSCs. It is interesting to note that in larvae, ABLKs act as interneurons in avoidance responses (Imambocus et al. 2022, 2025; K. Li et al. 2023) as they do in the adult wing defense in females (C. Liu et al. 2020), yet the behaviors differ mechanistically with crawling/turning in larvae and wing movement in adults.

## 6 Larval and Adult Efferent Neurons (or NSCs) of the Posterior Abdominal VNC and Their Functions

The third type of peptidergic VNC neurons dealt with here are neurons with cell bodies located more medially in the posterior portion of the abdominal neuromeres of the VNC that supply axons via a fused median nerve to the hindgut and associated structures in larvae and adults (median posterior efferents in Figures 4A and 6E). In adults, some of these neurons furthermore innervate the reproductive tract. Note that the functional classification of these neurons is less distinct. Some may be NSCs, others may be efferent neurons acting on the targets they innervate. So far, a hormonal action has actually been demonstrated only for the PDF‐expressing neurons in adults (Talsma et al. 2012) and PDF and ILP7 neurons in larvae (Linneweber et al. 2014).

In larvae, sets of efferent neurons have been identified that express AstA, ITP, PDF, CCAP, proctolin, MIP, and ILP7 (Anderson et al. 1988; Dircksen et al. 2008; Hergarden et al. 2012; Karsai et al. 2013; Miguel‐Aliaga et al. 2008; Santos et al. 2007; Taylor et al. 2004; Yoon and Stay 1995; W. Zhang et al. 2014; Figure 7A,B). It is not clear whether ITP, AstA, and CCAP are colocalized in larvae, as they are in adults (Figure 7A,C), and RYamide‐producing NSCs have not been described in larvae. Most of these NSCs/efferents can also be seen in adults, and some novel neurons have been added as follows.

The three pairs of abdominal PDF neurons in larvae co‐express glutamate and innervate the posterior hindgut, and together with another set of motor neurons (HGN1), they control defecation, primarily by means of colocalized glutamate (W. Zhang et al. 2014). Another study implicated PDF and ILP7 from posterior efferent neurons in nutrient‐dependent hormonal regulation of tracheal growth in larvae (Linneweber et al. 2014).

In adults, ITP, AstA, and CCAP are colocalized in two pairs of efferents, and one pair produces AstA alone (Gera et al. 2025), and another one RYamide (Veenstra and Khammassi 2017; Figure 7C). In adults, only two pairs of adult NSCs/efferents reproducibly produce PDF (Helfrich‐Förster 1997; Nässel et al. 1993; Talsma et al. 2012; Figure 7D), and the complement of ILP7‐producing neurons is diversified (Figure 7E,F). In adult females, there are 14 ILP7 efferents and in males 10 (Castellanos et al. 2013). These are of different types, and some are sex‐specific. In both sexes, there are three pairs of embryo‐derived cells that express only ILP7 and which innervate the hindgut. In adult males, two pairs of efferents, born post‐embryonically, express ILP7, glutamate, and serotonin and innervate the male reproductive tract (seminal vesicle) (Castellanos et al. 2013). In females, four pairs of adult ILP7 efferents can be seen. Two pairs correspond to the male adult neurons but express only ILP7 and glutamate, and two are found only in females and also express ILP7 and glutamate (Castellanos et al. 2013). The adult female efferents innervate oviduct muscle. Thus, the adult‐specific ILP7 neurons regulate reproductive processes. This has been primarily studied in female insects, where the post‐embryonic ILP7 neurons regulate ovulation and oviposition (Gou et al. 2014; Su et al. 2025; C. H. Yang et al. 2008). Interestingly, the female ILP7 neurons signal with glutamate to modulate contractions in the lateral oviducts to enable smooth passage into the common oviduct, whereas ILP7 peptide acts further down the oviduct to modulate oviposition (Su et al. 2025).

The abdominal PDF neurons that supply axons to the hindgut release PDF into the circulation to regulate contractions in the ureters of the Malpighian tubules (Talsma et al. 2012) and thereby influence secretion in the tubules (osmoregulation). The efferent RYamide neurons supply axons to the rectal papillae and might control water reabsorption (Veenstra and Khammassi 2017). Also, the efferent ITP neurons innervate the posterior hindgut, and the ITP receptor Gyc76C is expressed in rectal papillae suggesting a possible role for ITP in water reabsorption (Gera et al. 2025); the colocalized NPHs AstA and CCAP were not further investigated.

## 7 Further Examples of Functions of Specific Sets of Neurons in Larvae and Adults: Similarities and Differences

This section provides further examples of NPH signaling that differs between larvae and adults in prominent ways (Sections 7.1 and 7.2) or display substantial similarities (Sections 7.3 and 7.4). One set of peptidergic neurons controls ecdysis‐related processes (e.g., controlling muscle activity) in larvae and pupae but is part of totally different functions in adults (7.1). Another set of neurons form the circuits that constitute the biological clock, and this is rudimentary in larvae but extensive and complex in adults (7.2). The EECs of the intestine produce the same types of NPHs in both stages, although functions are mostly known only in adults (7.3). Finally, NPHs involved in regulation of food seeking and feeding are numerous in both stages, although mechanisms or peptide action may vary (7.4). I keep the first three sections short since there is an imbalance in the amount of data available for the two stages.

### 7.1 NPHs in the Ecdysis Cascade During Development and What They Do in Adults

A neuronal circuitry that has received substantial attention in larvae is formed by a relatively small number of peptidergic neurons that regulate ecdysis motor behavior and some related processes (summarized in Baker et al. 1999; Ewer and Reynolds 2002; Y. ‐J. Kim et al. 2006; McNabb et al. 1997; Truman 2005; B. H. White and Ewer 2014; Zitnan and Adams 2005; Žitňan et al. 2007). This set of neurons is located segmentally in the different neuromeres of the CNS, mainly in the VNC (Figure 8A), and activity in the cascade is triggered by the peptide ecdysis‐triggering hormone (ETH) released from peritracheal cells (also named Inka cells) located segmentally in the main tracheal trunks of the body (Y. ‐J. Kim et al. 2006; Zitnan et al. 1996). Additional to ETH, there are six NPHs in neurons of the CNS that have been implicated in this cascade of interactions: EH, FMRFamide, CCAP, LK, MIP, and bursicon (Figure 8B). These NPHs sequentially provide activation, inhibition, or modulation of the motor activity that underlies the shedding of the cuticle but also in, for instance, LK in tracheal clearing and bursicon in cuticle tanning (Dewey et al. 2004; C. W. Luo et al. 2005; see Table 1 for specific functions of the NPHs). It should be noted that there are other NPHs (e.g., AstA, CRZ, and PTTH) released from other neuron types that also influence ecdysis processes (see Tables 1 and 2), but here I want to emphasize the set shown in Figure 8. Of note is that a few neurons in the ecdysis‐regulating set in the VNC also have some other functions between molts (Figure 8C): Abdominal neurons deploy LK and MIP to modulate locomotion and light avoidance (Imambocus et al. 2022; Okusawa et al. 2014), and bursicon influences growth (Kubrak et al. 2024) and innate immune responses (An et al. 2012).

**FIGURE 8:**
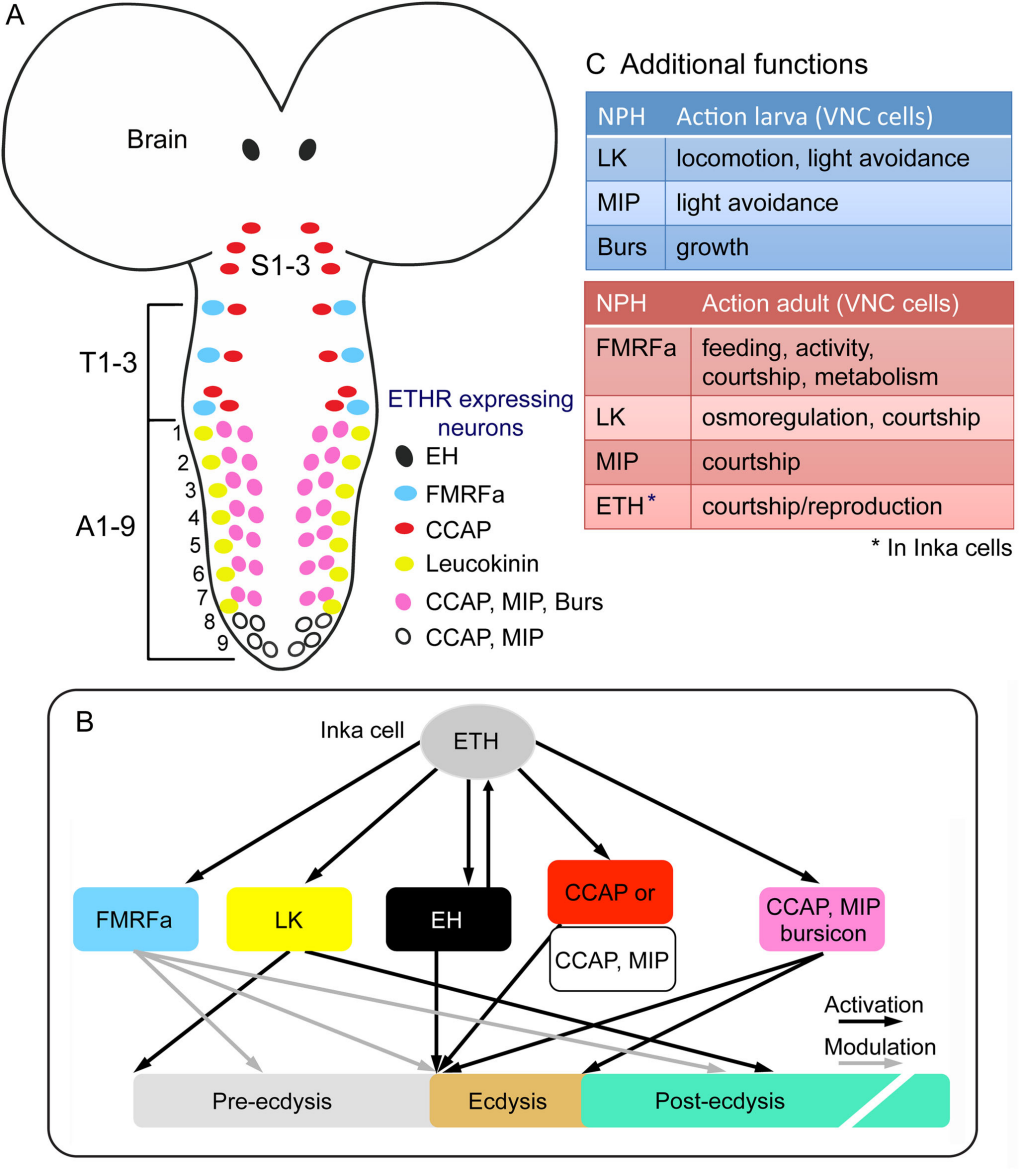
Peptidergic neurons that regulate ecdysis during development display other functions in adults. (A) A set of neurons in the brain, subesophageal zone (S1–3), and ventral nerve cord (T1–3 and A1–9) has been extensively investigated for its role in a cascade of events underlying the shedding of cuticle during stages of ecdysis and post‐ecdysis (shown in B). These neurons express the receptor of ecdysis‐triggering hormone (ETH), ETHR‐A, and produce EH, FMRFamide (FMRFa), crustacean cardioactive peptide (CCAP), LK myoinhibitory peptide (MIP), and bursicon (Burs). (B) ETH released from Inka cells (peritracheal cells), under ecdysone control, acts to regulate activity in the other peptidergic neurons that express ETHR‐A receptor. These neurons and their NPHs in turn influence motor activity and other functions during the ecdysis cascade to ensure timely shedding of cuticle and processes after ecdysis including glue expulsion to attach pupae and tanning of the cuticle. (C) Other functions of NPHs utilized by the same set of neurons in larvae and adults. (A and B) are originally based on Y. ‐J. Kim et al. (2006) but redrawn in Nässel and Zandawala (2019) and updated after Zitnan et al. (2026). For data and references in (C), see Tables 2 and 3.

While most of the neurons depicted in Figure 8A,B survive metamorphosis (the EH neurons do not, and CCAP neurons are fewer), their functions in adults are obviously not associated with development. Actually, only four sets of neurons corresponding to the ones shown in Figure 8A,B have been functionally characterized in adults (Figure 8C): the ones producing FMRFamide, LK, MIP, and ETH. Interestingly, all four of these NPHs regulate aspects of courtship/reproduction in adults (J. Chen et al. 2025; Deshpande et al. 2019; Y. H. Jang et al. 2017; S. S. Lee and Adams 2021; C. Liu et al. 2020; Meiselman et al. 2017; Meiselman, Ganguly, et al. 2022; Sadanandappa et al. 2026; Shao et al. 2019; see Table 3 for details). Additionally, LK from adult ABLKs acts as a diuretic hormone and modulates stress responses and feeding (Radford et al. 2002; Zandawala et al. 2018), and FMRFamide from Tv neurons plays roles in feeding, metabolism, and organismal activity (Lenz et al. 2015; Miyamoto et al. 2024; T. Song et al. 2023). Thus, like many of the NPHs of NSCs (and other neurons), these NPHs play roles in development in larvae and in reproduction in adults.

### 7.2 Clock Neurons in Larvae and Adults

The clock system is a good example of an extensive difference in neuronal wiring complexity in brain circuits of larvae and adults. Looking at numbers of primary clock neurons in the brain of *Drosophila*, it develops from 18 in the larva to about 242 in adults (Reinhard et al. 2024; Wegener et al. 2026; Figure 9). This is accompanied by a large difference in the number of NPHs utilized by clock neurons. In larvae, only PDF in the lateral neurons (termed LNv in larvae but survive metamorphosis and correspond to s‐LNv in adults) has been clearly identified. However, CCHamide1 (CCHa1) has been indicated indirectly in DN1a neurons (Acklin et al. 2026; Poe et al. 2024; see below), whereas in adults, 12 different NPHs are known in different expression patterns in multiple neuron types (Figure 9). I will present the larval clock circuit in some detail since it has not been reviewed in detail recently, but I keep the adult part brief while it has been extensively summarized recently with respect to NHPs (see, e.g., Ma et al. 2021; Muraro et al. 2013; Reinhard et al. 2024; Wegener et al. 2026).

**FIGURE 9:**
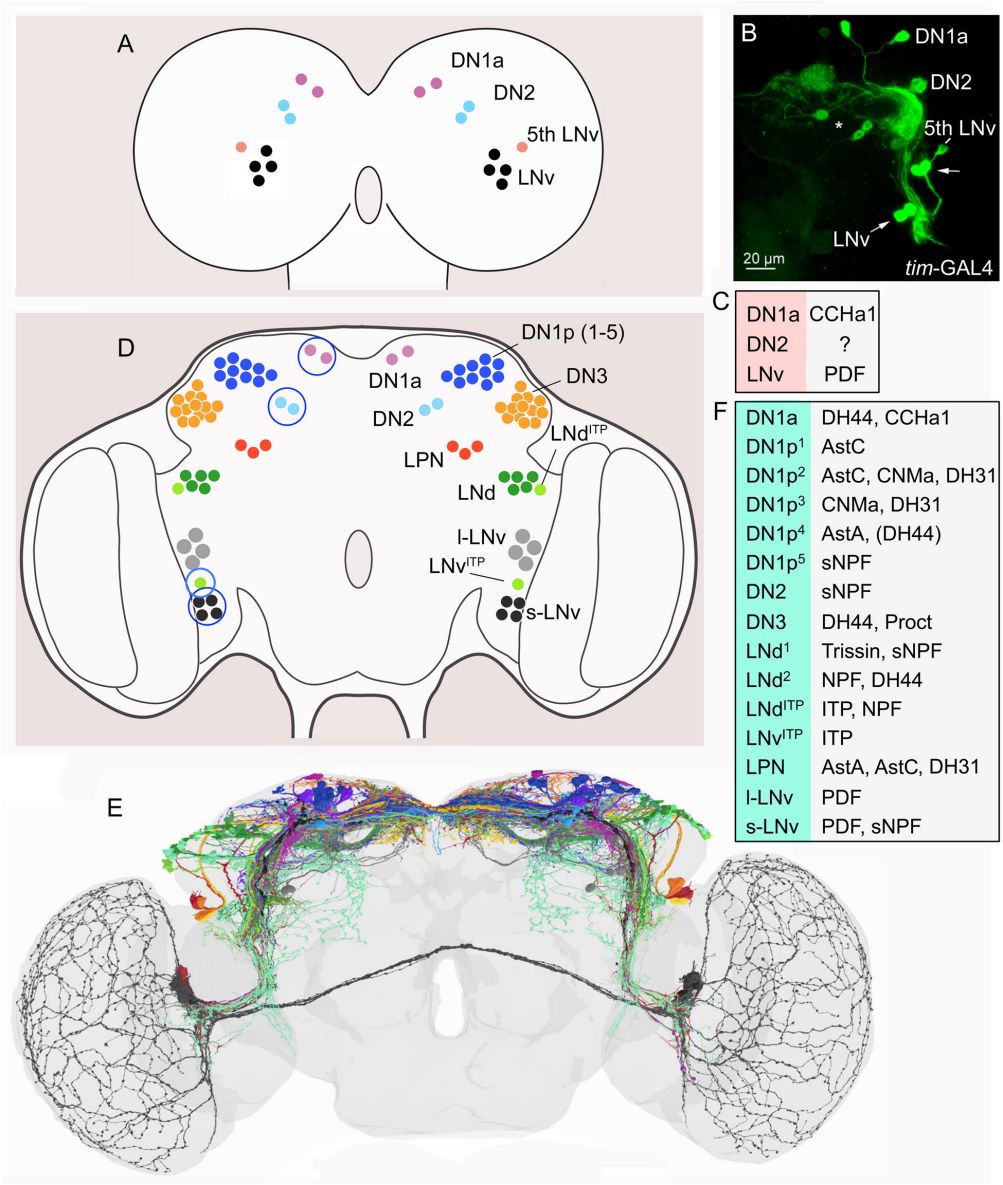
A drastic expansion of the clock circuits is seen in the numbers of clock neurons and NPHs at the larval‐to‐adult transition in *Drosophila*. (A) The clock neurons in the larval brain. Only four types of clock neurons have been identified. (B) Larval clock neurons identified with a *timeless* (*tim*)‐GAL4 driver. (C) Only PDF has been clearly demonstrated in larval clock neurons, whereas CCHa1 is likely present in DN1a neurons based on knockdown experiments (Acklin et al. 2026; Poe et al. 2024). In the 5th‐LNv, the peptide remains unknown (not shown in figure). (D) Adult clock neurons. The neurons encircled in blue are found both in larvae and adults (note that not all the neurons in the DN2 and DN3 clusters are shown). The LNv^ITP^ are also known as 5th‐LNv. (E) The adult clock neurons from the clock connectome. Note that the colors of the neurons in this panel do not always correlate with those in (D). (F) The different NPHs in adult clock neurons (further peptides have been indicated by single ‐cell transcriptomics, Reinhard et al. 2024, but need further confirmation). See the list of acronyms for the NPHs. (A) is redrawn from Wegener et al. (2026), (B) is from Hamasaka and Nässel (2006) with permission, (D) is redrawn from Reinhard et al. (2024), and (E) is from Reinhard et al. (2024; *Nature Communications* 15, 10392; open access).

In spite of its simplicity, the larval clock network can be entrained by light and drives circadian processes, including arousal, sleep, and circadian variation in light sensitivity, and it is integrated into the larval visual system and thus contributes to light sensing (phototaxis/light avoidance) and simple vision, as well as visual learning (see, e.g., Gerber et al. 2004; Justice et al. 2012; Larderet et al. 2017; Mazzoni et al. 2005; Poe et al. 2023; Yamanaka, Romero, et al. 2013). The larval clock also contributes to timing developmental transitions (ecdysis) via PTTH neurons and ecdysone (see Wegener et al. 2026).

The larval clock network consists of two types of photoreceptors (blue‐ and green‐sensitive) in the simple pair of eyes, the Bolwig organs, and four types of clock neurons, LNv, 5th‐LNv, DN1a, and DN2 (Figure 9A,B). Axons from the 12 photoreceptors in each eye connect to the LNv dendrites in the larval optic neuropil, where other neurons also converge (Helfrich‐Förster et al. 2002; Larderet et al. 2017). The circuits furthermore include neurons that do not express clock genes: second‐order neurons and modulatory neurons, several of which are engaged in the visual functions, others in the operation of the clock (Larderet et al. 2017). Connectomics of the visual system of first instar larvae established a total of (in one hemisphere) 11 second‐order neurons, two third‐order neurons, one serotonergic neuron, and a midline pair of octopaminergic neurons innervating larval optic neuropils in both hemispheres (Larderet et al. 2017). These neurons are all associated with the larval optic neuropil. A recent study furthermore showed that CCHa1 knockdown in DN1a neurons affects MNCs that express DH44; this circuit drives diurnal sleep rhythms starting in early third instar larvae (Acklin et al. 2026; Poe et al. 2023). Before that stage, arousal and larval sleep are not under clock control.

An important role of the clock circuits in larvae (and later in the evolved clock of pupae and pharate adults) is in developmental timing. The clock drives activity in IPCs and PTTH neurons to regulate ecdysone production in the prothoracic gland and thus ecdysis, including final eclosion (see Wegener et al. 2026). Here, it can be noted that a set of PDF‐producing neurons (PDF‐Tri), which do not express clock genes, can be seen in the tritocerebrum of pharate adults (Selcho et al. 2018; Figure 2E). These neurons are involved in the regulation of eclosion at the pupa–adult transition and are found neither in larvae nor mature adults (Selcho et al. 2018). A similar set of transient interneurons (DM^EH^ and Dl^EH^) was found in two sites of the protocerebrum; these signal with EH to regulate eclosion (Scott et al. 2020; Figure 2E).

The larval clock neurons overlap functionally with components of the larval visual system, and it has been found that the LNv clock neurons utilize PDF to mediate the circadian signals, whereas the rapid light responses are not dependent on PDF (or clock genes, like cryptochrome) but likely an SMN (Mazzoni et al. 2005). The phototaxis behavior switches to light avoidance toward the end of the larval life (non‐feeding stage), and this change is mediated by PTTH and ecdysone that concomitantly regulate ecdysis (Yamanaka, Romero, et al. 2013). PTTH acts both on the Bolwig photoreceptors and peripheral Class IV multidendritic neurons in the body wall to induce this sensitivity switch.

As mentioned, the adult clock circuits are far more complex (Figure 9D,E), and the complement of neuropeptides in clock neurons is increased from one to 12 (Figure 9C,F; and several additional NPHs suggested from single‐cell transcriptomics—Reinhard et al. 2024—that require further confirmation). Importantly, there are multiple cases where two or more NPHs are colocalized in clock neurons (Figure 9F; Abruzzi et al. 2017; Johard et al. 2009; Ma et al. 2021; Reinhard et al. 2024). A few components persist from the larval clock. Four photoreceptors originating from the Bolwig organ are in adults located in the extra‐retinal eyelet near the optic lobes, and their axons connect to dendrites of the s‐LNv in the so‐called accessory medulla (Helfrich‐Förster et al. 2002). Furthermore, the LNv, DN1a, DN2, and 5‐th LNv also survive metamorphosis (Figure 9A,D; see Wegener et al. 2026) and integrate into the complex adult circuitry. The literature on adult clock circuits and functions is extensive (see recent reviews, Reinhard et al. 2024; Wegener et al. 2026) and in very brief the clock controls behavioral and physiological rhythms of different kinds. These include activity rhythms and sleep, and regulation of neuroendocrine systems that control feeding, metabolic homeostasis and reproduction (including sex pheromone production and egg laying), and memory formation (Kurata et al. 2026; Liang et al. 2023; Reinhard et al. 2024; Riva et al. 2026; Wegener et al. 2026). In this context, it is of interest to note that PDF serves important and distinct functions in the clock of larvae and adults, respectively, and that the increased complexity in adults involves the addition of the adult‐specific PDF‐producing l‐LNv cells with functions distinct from those of the adult s‐LNv cells. Thus, two sets of neurons in the same adult network use the same NPH and yet have distinct roles in the circuitry.

The clock circuitry operates both by means of synaptic connections with SMNs, including monoamines, and non‐synaptically with neuropeptides (Ahmad et al. 2021; Kurata et al. 2026; Reinhard et al. 2024). The latter wire‐less circuitry is incompletely mapped and needs further study. However, some functional connections can be inferred by mapping neuropeptide receptors to presumptive target neurons and subsequent experimental testing (see Fukuda et al. 2025; Reinhard et al. 2024). Interestingly, a recent study showed that the s‐LNv neurons are not synaptically connected to IPCs but signal to them with sNPF and PDF by means of volume transmission over a distance of 15–20 µm (Hameed et al. 2026).

It is of note that the 12 NPHs utilized by clock neurons are also important components of other neuron types (Figure 10). These include NSCs of the brain and VNC, as well as different types of interneurons, efferents and gut EECs. For instance, AstA, ITP, PDF, and proctolin are expressed both by clock neurons and efferents innervating the gut. As another example, DH31 is additionally expressed by LNCs, local brain interneurons, NSCs in the VNC, and gut EECs. This again emphasizes that NPH actions are diverse and not necessarily functionally coupled.

**FIGURE 10:**
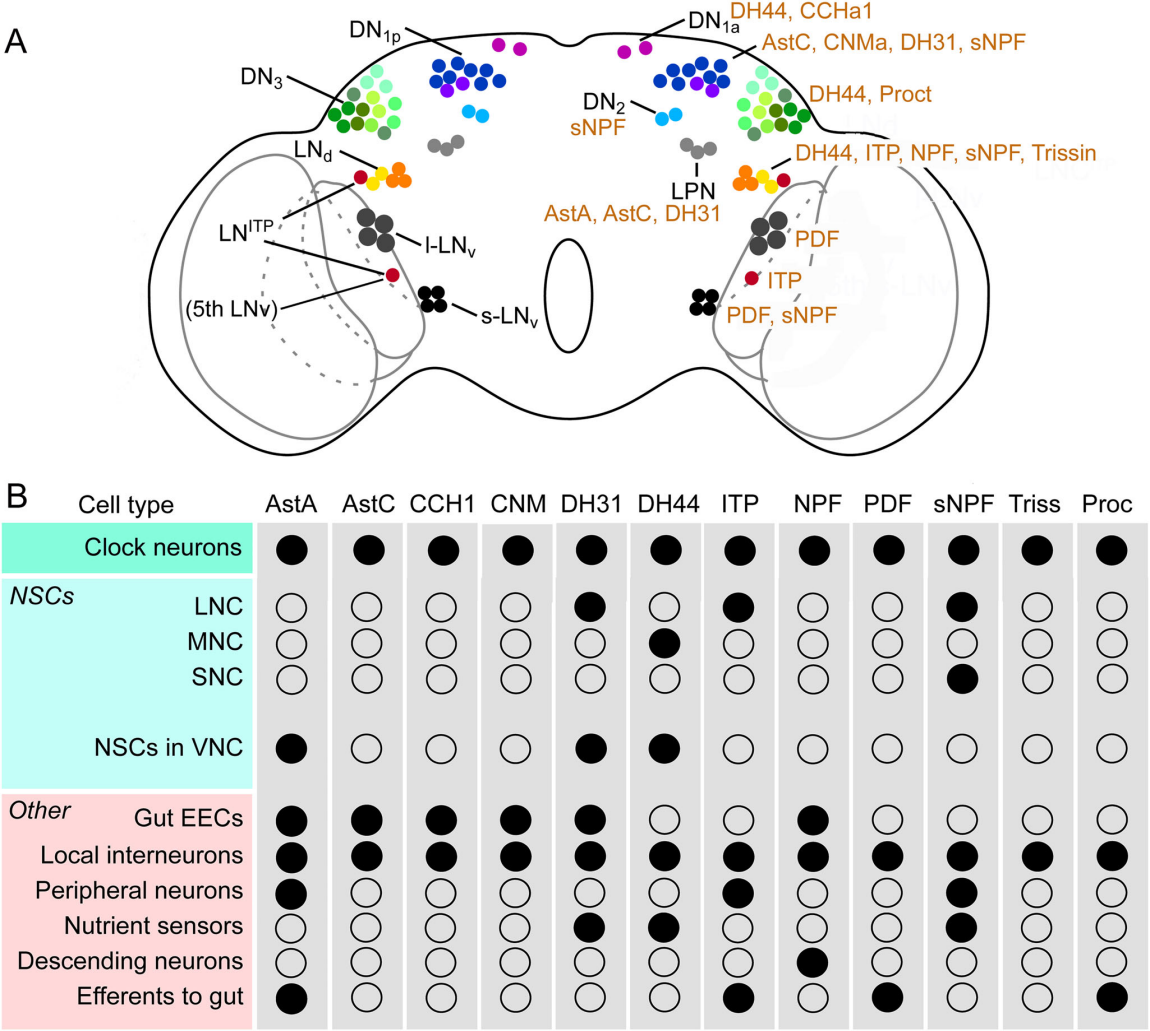
In adult flies, NPHs of clock neurons are also produced by other cell types. (A) Clock neurons in the adult *Drosophila* brain (from Reinhard et al. 2024), with permission, *eLife*, open access. The NPHs produced in the clock neurons are shown in red (peptide acronyms in list). (B) NPHs of clock neurons are produced by multiple other cell types. This figure is from Nässel (2025), with permission.

### 7.3 Gut Endocrine Cells in Larvae and Adults

In recent years, endocrine cells (EECs) of the intestine and their roles in interorgan signaling have seen an increase in interest (summarized in Miguel‐Aliaga et al. 2018; Rewitz 2026). NPHs released from EECs play multiple hormonal roles in physiology and behavior, including regulation of feeding, selection of macronutrients, metabolic homeostasis, reproduction, nutrient‐dependent sleep, and immune responses but also act locally to modulate, for example, gut peristalsis and lipid metabolism (see LaJeunesse et al. 2010; Miguel‐Aliaga et al. 2018; Rewitz 2026; W. Song et al. 2014; Veenstra et al. 2008). EECs can be nutrient‐sensing or respond to other factors (including circulating NPHs), and they signal to the brain to regulate appetite, metabolic homeostasis, immune responses, and behavior. Thus, the intestine is an important interface between the body and the brain and also between the external environment and the internal physiology.

Since there are very few publications describing functions of NPHs in larval EECs, I will restrict this section to a comparison of NPH distribution and a brief summary of mainly adult functions. It is important to note that the midgut, where most EECs reside, undergoes histolysis during metamorphosis and thus the adult EECs emerge *de novo* (S. Jang et al. 2021; Miguel‐Aliaga et al. 2018). Yet the same 11 NPHs are present in EECs of both stages (Figure 11A–C; S. Jang et al. 2021; Miguel‐Aliaga et al. 2018; Reiher et al. 2011). Additionally, a recent paper identified the peptide limostatin (Lst) in EECs of the anterior midgut of larvae (Bai et al. 2026; Figure 11C).

**FIGURE 11:**
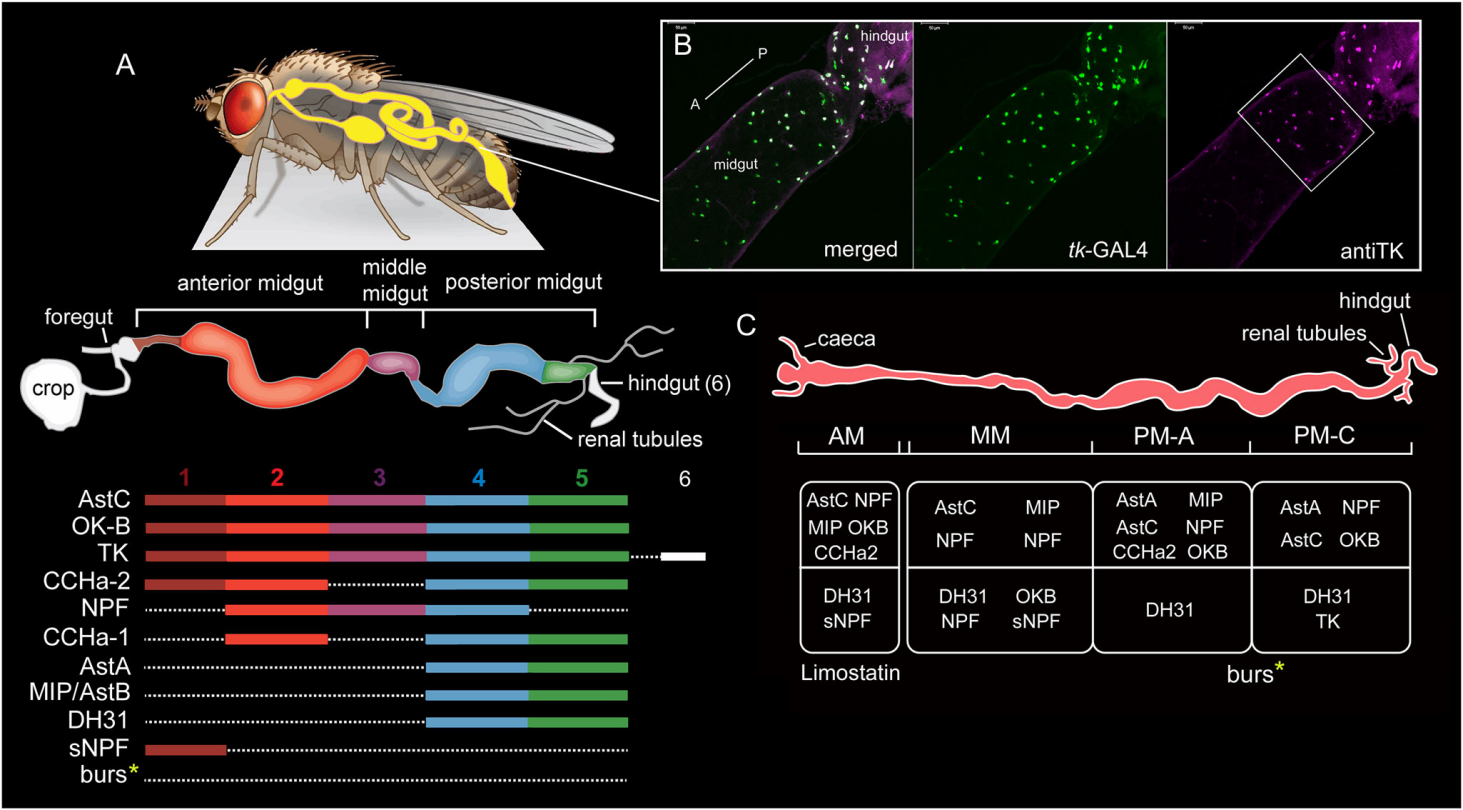
Intestinal enteroendocrine cells (EECs) in larvae and adults of *Drosophila*. Note that the renal tubules are shown truncated in both stages. (A) The adult intestine and its complement of peptidergic EECs. Ten peptides (NPHs) have been identified (see acronyms in separate list) and are distributed in the five main regions of the midgut (1–5). One peptide, tachykinin (TK), is also found in EECs in the anterior hindgut (region 6; also shown in B). Note that the NPHs are distributed in different patterns in the different regions, and several are co‐expressed in the same EECs (not detailed here). (B) Immunolabeling with anti‐tachykinin (TK) and *tk*‐GAL4 expression in the posterior midgut and anterior hindgut of adult *Drosophila*. (C) Larval EECs express the same NPHs but in slightly different distribution patterns, compared to adults, and one additional peptide, limostatin, was identified in the anterior midgut. The midgut is divided into anterior (AM), median (MM) and two posterior regions (PM‐A and PM‐C). The distribution of NPHs in EECs is shown schematically in the boxes where co‐expression patterns are indicated by the grouping of acronyms. One major difference is the very restricted distribution of TK in the larva, compared to the adult. No NPH has been identified in the larval hindgut. (A) is modified from Nässel and Zandawala (2022), (B) is from Liu, Luo, and Nässel (2016) with permission, (C) is redrawn from S. Jang et al. (2021).

In larvae, peptidergic EECs are only found in the midgut, and the different NPHs are distributed in different regions, often colocalized in the same cells in different patterns (Figure 11C). NPHs released from larval EECs have been implicated in regulation of intestinal stem cell proliferation (bursicon; Scopelliti et al. 2014b), appetite and developmental timing (CCHa2; Ren et al. 2015), amino acid sensing (DH31; J. H. Park et al. 2016), immune responses (DH31; Tleiss et al. 2024), gut peristalsis (DH31; LaJeunesse et al. 2010), and regulation of ecdysone production in the prothoracic gland (NPF; Kannangara et al. 2020) and dietary amino acid‐dependent inhibition of ILP secretion from IPCs to regulate developmental plasticity (Lst; Bai et al. 2026; see also Table 2). Since only five out of eleven EEC NPHs have been studied functionally in larvae so far, it can be expected that further functions will be discovered. It is, for instance, a bit surprising that only bursicon, CCHa2, and NPF have been found in regulation of development; an extended screen of developmental roles of EECs needs to be performed.

In adult flies, the 11 NPHs are found in EECs of the midgut, and TK is additionally found in the anterior hindgut (Figure 11A,B). Also in adults, the NPHs are expressed in different regions of the midgut, in many cases colocalized with one or more other NPHs (Miguel‐Aliaga et al. 2018; Reiher et al. 2011; Veenstra et al. 2008). The regional distribution of NPHs is similar but not identical to that in larvae (Figure 11A,C). In addition to the EEC‐associated NPHs, CNMamide is expressed in enterocytes of the midgut (B. Kim et al. 2021) and ILP3 in midgut muscle cells (Veenstra et al. 2008).

In adults, nine of the 11 NPHs of EECs have been assigned multiple roles in physiology and behavior (Tables 3 and 4); only MIP and orcokinin‐B remain to be investigated. The NPH functions discovered so far include regulation of nutrient sensing (AstC, bursicon, CCHa2, DH31, NPF), food seeking and feeding (AstA, AstC, NPF), activity and sleep (CCHa1), nutrient‐dependent oogenesis (NPF), metabolic homeostasis (AstA, AstC, bursicon, NPF), osmoregulation (DH31, NPF, TK), gut function such as defecation peristalsis, lipid metabolism, immune response (DH31, TK), and stress responses (DH31); for references see Tables 3 and 4. Finally, CNMamide in amino acid sensing midgut enterocytes regulates appetite (B. Kim et al. 2021) and specifically amino acid appetite (via DH44 neurons; B. Kim et al. 2026). Similar EEC‐associated NPH functions in the two stages have been shown only for CCHa2 in appetite (Ren et al. 2015) and DH31 in immune responses (Benguettat et al. 2018; Tleiss et al. 2024).

### 7.4 NPHs Regulate Food Seeking and Feeding both in Larvae and Adults

Food seeking and feeding in larval and adult *Drosophila* differ. Larval food intake is almost continuous during wake and supports growth, development and “daily life physiology.” In adults, feeding is intermittent and primarily to support daily life physiology and some adult maturation. In adult females, feeding is additionally important for ovary and egg development and post‐mating physiology and behavior (Badisco et al. 2013; Itskov and Ribeiro 2013; Okamoto and Mizoguchi 2026). The brain receives signals from peripheral tissues (intestine and fat body) that report the internal metabolic state and central neurons interpret these to ensure maintenance of nutrient and energy homeostasis (Droujinine and Perrimon 2016; Itskov and Ribeiro 2013; Ko et al. 2015; Mahishi et al. 2024; Mahishi and Huetteroth 2019; Miroschnikow et al. 2020; Pool and Scott 2014; J. Zhao et al. 2025). NPHs play an important role in this interorgan signaling and the brain's response to hunger leading to appetitive behavior and induction of food seeking and feeding (Tables 3, 4, and 7). This includes peptidergic nutrient‐sensing cells, as well as NPH‐induced enhancement of food odor sensitivity and altered taste preferences, regulation of foraging, food intake, and satiety signaling (see reviews: Audsley and Weaver 2009; Itskov and Ribeiro 2013; Ko et al. 2015; S. Lin et al. 2019; Nässel and Zandawala 2019; Pool and Scott 2014; J. Zhao et al. 2025; Tables 3, 4, and 7). Some NPHs act in arbitration between feeding and other competing behaviors (see, e.g., H. ‐H. Lin et al. 2022; Martelli et al. 2017; Nässel and Zandawala 2022; Schlegel et al. 2016). In the following, I briefly summarize roles of NPHs in aspects of larval and adult feeding but do not consider NPHs that regulate digestion, gut function, and other post‐feeding mechanisms (e.g., diuresis, defecation, nutrient storage/allocation, and bona fide metabolism). Figure 12 shows the different NPHs and neurons/NSCs that regulate aspects of foraging and feeding in larvae and adults. These NPHs are also comprehensively listed in Tables 3, 4, and 7 (with references). The NPHs that regulate foraging and feeding in both larvae and adults are AKH, AstA, CRZ, DH44, DILP2, 3, 5, Hugin‐PK, NPF, and sNPF.

**TABLE 7:** Neuropeptides/peptide hormones regulating nutrient sensing, food seeking, and feeding via larval and adult brain NSCs and other neurons.

**Peptide**	**Cell type**	**Role**	**References**
**LARVAE**	**NSC**		
DH44	MNC	Glucose sensing	(Poe et al. 2024)
CRZ	LNC	Fructose sensing	(Kulshrestha et al. 2024; Mishra et al. 2013)
Hugin	SEZ	Food avoidance	(Surendran et al. 2017)
		Food search/feeding	(Hückesfeld et al. 2016; Schlegel et al. 2016; Schoofs et al. 2014)
AKH	CC	Glucose sensing	(J. Kim and Neufeld 2015; S. K. Kim and Rulifson 2004)
**LARVAE**	**Other cells**		
DH31	Gut EECs	Amino acid sensing	(J. H. Park et al. 2016)
AstA	?	Foraging	(C. Wang et al. 2012)
CCHa2	Gut EECs and brain neurons	Appetite	(Brunßen and Suter 2024)
NPF	Brain neurons	Feeding decision	(de Tredern et al. 2025)
		Food odor/appetite	(Pu et al. 2018; Y. Wang et al. 2013)
		Food search, olfaction	(Rohwedder et al. 2015)
**ADULTS**	**NSC**		
DH44	MNC	Food search/feeding	(Dhakal et al. 2022; Dus et al. 2015; Oh et al. 2021; Z. Yang et al. 2018)
		Amino acid sensing	(Z. Yang et al. 2018)
CRZ	LNC	Food search/feeding	(Kubrak et al. 2016)
ILPs	MNC	Food search/feeding	(González Segarra et al. 2023; X. Li et al. 2024; Owusu‐Ansah and Perrimon 2014)
DMS	MNC	Food search/feeding	(Hadjieconomou et al. 2020)
DSK	MNC	Feeding	(Söderberg et al. 2012)
Hugin	SEZ neurons	Food search/feeding	(King et al. 2017; Melcher and Pankratz 2005; Schlegel et al. 2016)
ITP	LNC	Feeding	(Galikova et al. 2018; Gera et al. 2025)
AKH	CC	Food search/feeding	(Bharucha et al. 2008; Gáliková et al. 2015; G. Lee and Park 2004; Pauls et al. 2021; Yu et al. 2016)
**ADULTS**	**Other cells**		
AstA	Brain neurons or EECs	Food search/feeding	(J. Chen et al. 2016; Hentze et al. 2015; Hergarden et al. 2012; Tsao et al. 2018; C. Wang et al. 2012)
AstC	EECs		(Kubrak et al. 2022)
AstC	Brain neurons		H. ‐H. Lin et al. 2022)
Bursicon	VNC neurons		(Kubrak et al. 2024)
CAPA	NSCs brain, VNC		(Hadjieconomou et al. 2020)
CCHa2	Gut, fat body		(Ren et al. 2015; Sano et al. 2015)
CNMa	EECs		(Hückesfeld et al. 2021)
DH31	EECs		(H. ‐H. Lin et al. 2022)
DMS	Brain neurons		(G. Wu et al. 2024)
DSK	IPCs, MP1/MP3		(D. Guo et al. 2021; X. Li et al. 2024; Nässel and Wu 2022; Söderberg et al. 2012)
FMRFa	VNC‐NSCs to gustatory receptors		(T. Song et al. 2023)
FMRFa	Brain neurons		(G. Wu et al. 2024)
Hugin	SEZ neurons		(King et al. 2017; Melcher and Pankratz 2005; Schlegel et al. 2016)
LK	Brain neurons		(Al‐Anzi et al. 2010; Zandawala et al. 2018)
MIP/AstB	Brain neurons		(Min et al. 2016)
NPF	Brain neurons		(Beshel et al. 2017; Chung et al. 2017; Corfas et al. 2019; de Tredern et al. 2025; Landayan et al. 2021; Pu et al. 2018; Shen and Cai 2001; Tsao et al. 2018; Y. Wang et al. 2013; Q. Wu, Zhang, et al. 2005; Q. Wu, Zhao, and Shen 2005)
NPF	Gut EECs		(Gao et al. 2024)
SIFa	Global interneurons		(Dreyer et al. 2019; Martelli et al. 2017; Y. Song et al. 2025)
sNPF	Brain neurons (multiple types)		(de Tredern et al. 2025; Hong et al. 2012; K. S. Lee et al. 2004; C. Liu et al. 2024; Root et al. 2011; Shen and Cai 2001; Tsao et al. 2018)
TK	Brain neurons (multiple types)		(Ko et al. 2015; Musso et al. 2021; Qi et al. 2021)

*Note:* Peptide abbreviations are in the list of acronyms.

**FIGURE 12:**
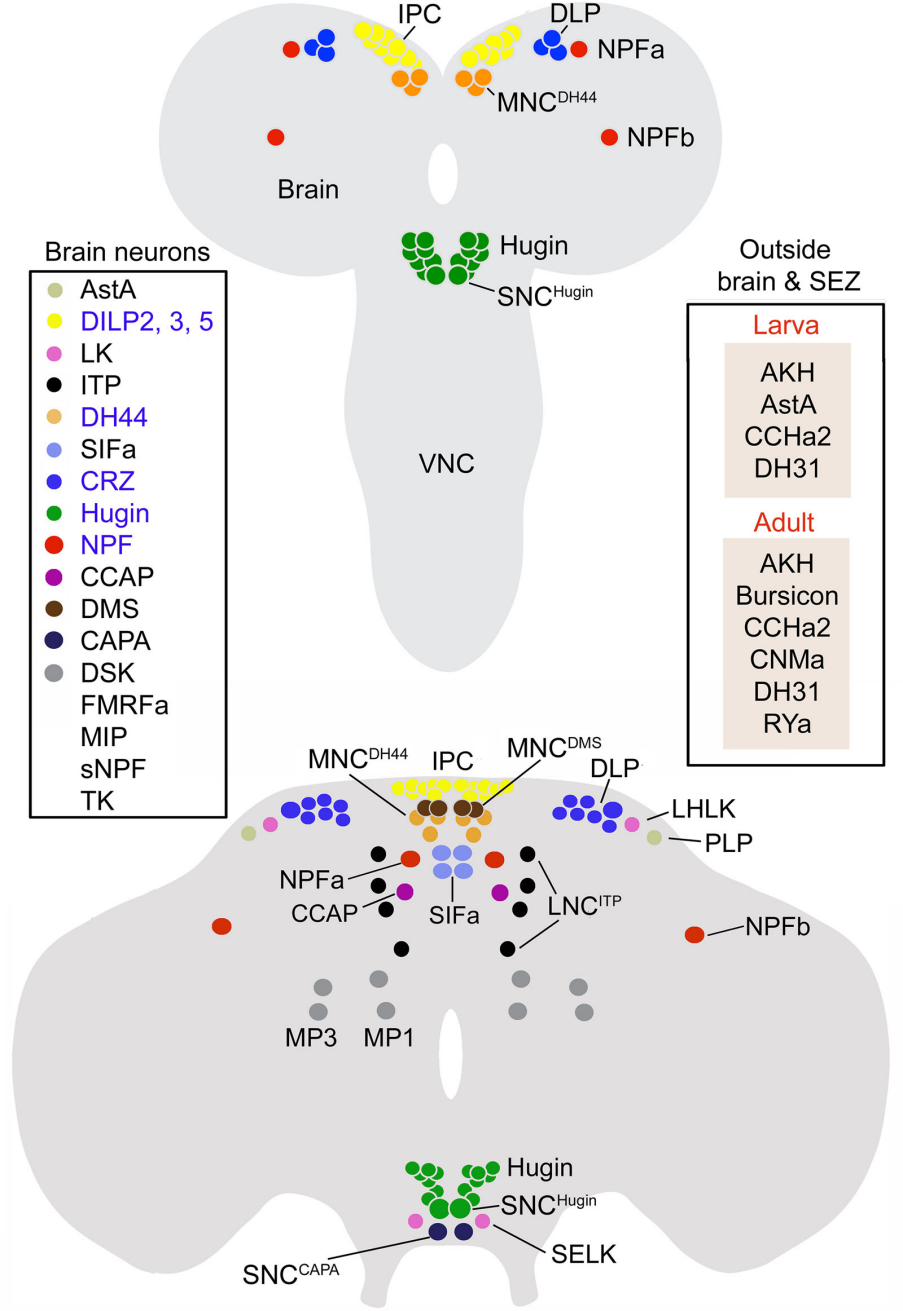
Neuropeptides/peptide hormones regulating nutrient sensing and/or food seeking and feeding in larval and adult *Drosophila*. Larval CNS (top) and adult brain (bottom) with brain and subesophageal zone (SEZ) neurons involved in nutrient sensing and/or feeding. Guide to NPHs located in brain and SEZ neurons is given in the box to the left. The peptides listed in the box to the right are located in cells outside the brain and SEZ (except for AKH, in the intestine), or in the case of AstA, it is not clear whether they are in cells of the brain or intestine. The peptides affecting feeding/nutrient sensing in both larvae and adults are indicated in blue font in the list with color‐coded cell bodies to the left. The brain neurons are insulin‐producing cells (IPC), dorsolateral neurons (DLP; also known as LNC^CRZ^), Median NSCs‐producing DH44 (MNC^DH44^), two types of NPF interneurons (NPFa and b) and a cluster of Hugin cells (note that these are a heterogeneous group, including a pair of NSCs, SNC^Hugin^). In the adult brain, several additional neurons are implicated CCAP, LHLK, MNC^DMS^, MP1, MP3, PLP, SIFa, SELK, and SNC^CAPA^. For references, see Tables 3 and 4.

AKH‐producing cells in corpora cardiaca are involved in glucose sensing in larvae (S. K. Kim and Rulifson 2004), and in adults, multiple studies have implicated AKH in regulation of taste, foraging, and feeding (see Tables 3 and 7 for references). In adults, starvation‐induced AKH release stimulates foraging (G. Lee and Park 2004; Pauls et al. 2021; Yu et al. 2016) and feeding (Gáliková et al. 2017).

AstA inhibits foraging and feeding in both larvae and adults. Note that in larvae, it is not clear which of the AstA cell types; it could be neurons of the CNS or gut EECs (C. Wang et al. 2012). In adults, AstA has received more attention (Tables 3 and 7). Specific brain AstA neurons (PLP) have been implicated as being downstream of the clock and involved in arbitration between sleep and feeding (J. Chen et al. 2016). Furthermore, one study implicated AstA neuron input to circuits of the mushroom bodies via dopaminergic neurons that induce reduced foraging (Tsao et al. 2018). Another paper suggests an indirect role of EEC‐derived AstA (Y. Li et al. 2023). That study shows that gut‐derived AstA acts on AKH cells to mobilize energy reserves during sleep deprivation and thus indirectly affects food intake.

CRZ‐expressing LNCs are fructose and glucose sensing in larvae (Kulshrestha et al. 2024; Mishra et al. 2013) and thus likely to affect feeding. In adults, the same CRZ neurons are also fructose sensing (Miyamoto et al. 2012) and do regulate feeding (Kubrak et al. 2016).

DH44‐expressing MNCs are glucose sensing in larvae (Poe et al. 2024), but no studies indicate direct involvement in feeding. In adults, on the other hand, multiple studies are available that implicate DH44‐expressing MNCs and VNC neurons (NSCs) in feeding. DH44‐expressing MNCs are sensing both sugar (Dus et al. 2015) and dietary amino acids (Z. Yang et al. 2018) and regulate metabolic state‐dependent feeding and crop function (Dhakal et al. 2022). These DH44 neurons receive inhibitory signals from crop distension via Piezo stretch receptors and also from Hugin‐expressing SEZ neurons (Oh et al. 2021). Furthermore, DH44 from the ABLKs in the VNC play a role in food intake, probably by hormonal action (Zandawala et al. 2018).

Hugin neurons of the SEZ and their circuitry have been studied in detail in larval arbitration between locomotion and feeding (Hückesfeld et al. 2016; Melcher and Pankratz 2005; Schlegel et al. 2016; Schoofs et al. 2014). In adults, the Hugin neurons also affect locomotion (King et al. 2017) and play a role in food intake via SIFamide‐expressing brain neurons (Martelli et al. 2017).

NPF plays important roles in larval feeding. These include regulation of motivation to feed (de Tredern et al. 2025), increasing food attraction, including modulation of odor sensation (Pu et al. 2018; Shen and Cai 2001; Y. Wang et al. 2013) and food reward (Rohwedder et al. 2015). NPF neurons are furthermore involved in food preference and avoidance of noxious food, under influence of ILP signaling (Q. Wu, Zhang, et al. 2005; Q. Wu, Zhao, and Shen 2005). In adults, NPF is also orexigenic and increases food attraction (Beshel et al. 2017) and furthermore acts as a hunger signal that triggers wakefulness (Chung et al. 2017). NPF neurons regulate thirst and hunger under control of AstA‐expressing neurons (Janu neurons) in the SEZ (Landayan et al. 2021). NPF additionally regulates food seeking via action on different dopaminergic neurons that are part of the circuitry of the mushroom bodies (Miroschnikow et al. 2020; Tsao et al. 2018). NPF derived from gut EECs has an anorexigenic effect. In mated females, amino acid‐sensing EECs release NPF that acts on dopaminergic neurons that via brain circuits inhibit feeding (Gao et al. 2024).

Although sNPF has been implicated in feeding in adults (de Tredern et al. 2025; Hong et al. 2012; Root et al. 2011; Tsao et al. 2018), no clear evidence is available in larvae, although sNPF acts on IPCs and ILP signaling to induce growth (K. S. Lee et al. 2008), which probably involves increased food intake.

As seen in Figure 12, the cell bodies of the peptidergic neurons that regulate feeding are not clustered in the same region, but distributed in different parts of the brain. This is especially obvious in the adult CNS. Here, the peptidergic NSCs are in the MNC, LNC, and SEZ clusters (SEZ‐NSC), and other peptidergic neurons spread out in various regions of the protocerebrum and SEZ. In the larval brain, only the four NPF neurons are outside the three NSC clusters. The peptidergic SEZ neurons are in both stages associated with gustatory neurons and neurons in the circuitry that regulates feeding motor programs and feeding motoneurons (Gera et al. 2025; Mahishi et al. 2024; Miroschnikow et al. 2020; Miroschnikow et al. 2018; Pool and Scott 2014; Savaş et al. 2026; Schlegel et al. 2016). Other feeding‐regulating neurons are either nutrient‐sensitive or receive inputs from sensory cells (see Tables 2, 4, and 7). As mentioned, the mushroom bodies also play a central part in regulation of feeding by coordinating hunger–satiety responses from peptidergic neurons that interact with dopaminergic neurons innervating the mushroom bodies (Tsao et al. 2018). Finally, peptidergic inputs (sNPF, TK, ILPs) to the antennal lobe circuitry, as well as olfactory sensory neurons, affect feeding by modulating sensitivity to food odors depending on hunger state and ILP signaling (Ko et al. 2015; Root et al. 2011).

Feeding and the metabolic/nutritional state play important roles in development and reproduction, and thus engage neurons in different (but overlapping) neuronal pathways in larvae and adults. This is summarized in Figure 13 (see also Kay et al. 2025). This figure shows how the same peptidergic brain neurons (producing AstA, CRZ, NPF, and ILPs) and gut EECs regulate feeding, insulin signaling, and downstream production of JH and ecdysone to affect growth and developmental timing in larvae and ovary maturation and reproduction in adults (females). Also, Hugin cells of the SEZ play roles in larval feeding and ecdysone production and adult feeding and JH production. Taken together, these findings suggest that some nutrient‐dependent NPHs switch functions from development to reproduction during the transformation from larva to adult. Cited literature is given in legend to Figure 13.

**FIGURE 13:**
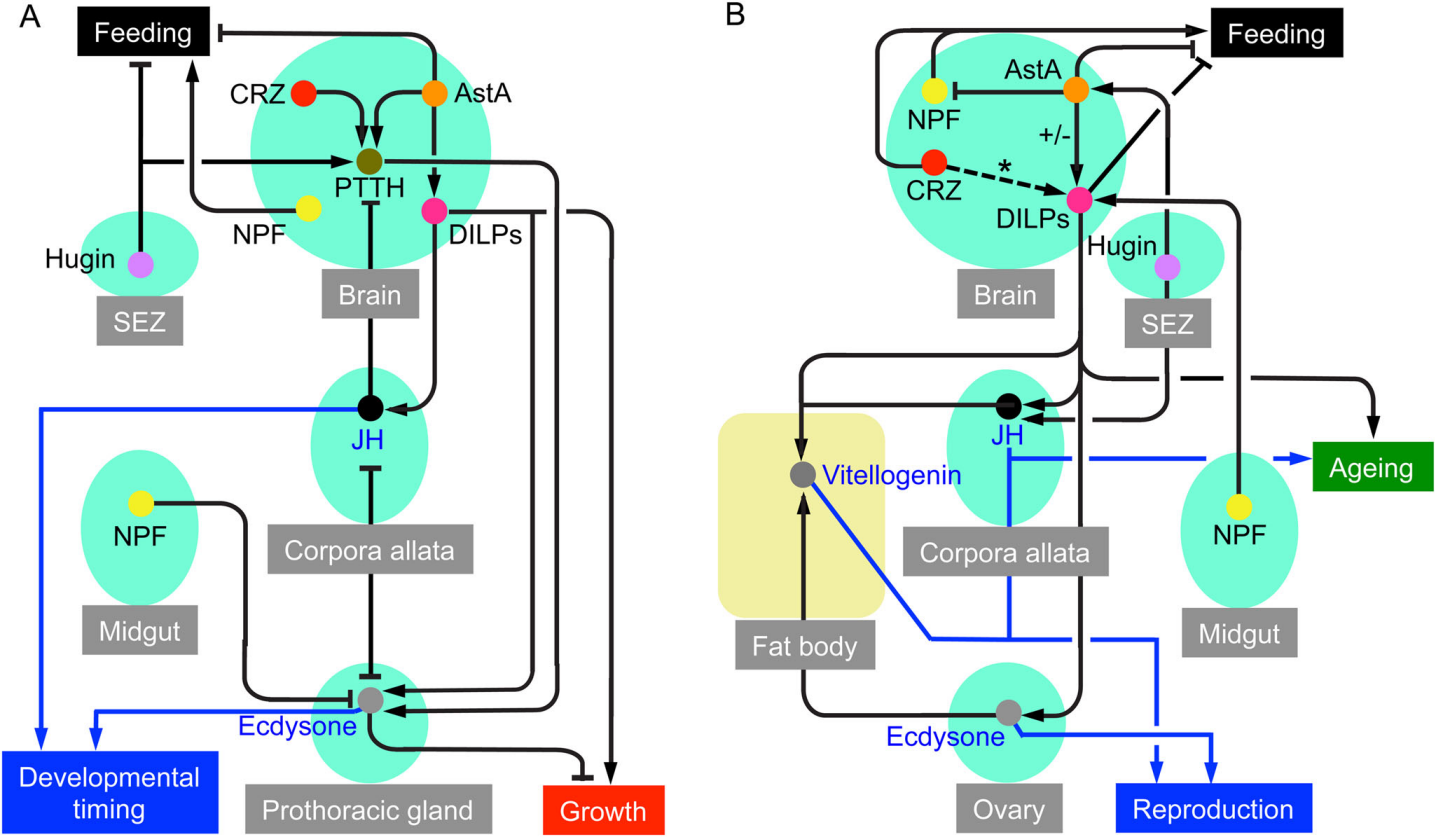
Peptidergic pathways regulating larval and adult functions in *Drosophila* related to feeding, development, growth, and reproduction. The peptides shown alter their functions between larvae and adults, except their roles in the regulation of food seeking and feeding (indicated as feeding in the figure). Both in larvae and adults feeding depends on nutrient sensing in the fat body, gut endocrine cells, or some specific brain neurons. Also, growth, ovary maturation, and reproduction are nutrient‐dependent. Note that here reproduction refers to female flies. (A) In the larva, a set of peptidergic neurons form pathways that regulate developmental timing or growth, dependent on nutrient status (and nutrient sensing). These include the brain, subesophageal zone (SEZ), corpora allata, prothoracic gland, and midgut (and fat body, not shown). Feeding is regulated by neuropeptide F (NPF; Pu et al. 2018; Y. Wang et al. 2013), allatostatin A (AstA; C. Wang et al. 2012), and Hugin pyrokinin (Hugin; Schlegel et al. 2016) and furthermore by insulin‐like peptides (DILPs) released from insulin‐producing brain neurons (Colombani et al. 2005) to inhibit NPF neurons in fed larvae. The role of CRZ in feeding has not been determined in larvae. The same peptides regulate developmental growth and timing (including molts) via regulation of juvenile hormone (JH) and ecdysone production in corpora allata and prothoracic gland, respectively. The ecdysone production is regulated by PTTH and DILPs released from brain neurons (McBrayer et al. 2007). These PTTH neurons are activated by Hugin‐PK, CRZ, and AstA (Deveci et al. 2019; Imura et al. 2020; Ohhara et al. 2024). JH and ecdysone mutually inhibit each other's biosynthesis (S. Liu et al. 2018). DILPs have direct effects on nutrient‐dependent tissue growth (Colombani et al. 2005; Demontis and Perrimon 2010; Ikeya et al. 2002). (B) In the adult, most of the same peptides regulate feeding and reproduction (including sexual maturation). This figure shows female pathways. NPF, CRZ, and AstA and indirectly Hugin (via sugar sensing), regulate feeding (Hentze et al. 2015; Hergarden et al. 2012; Kubrak et al. 2016; Oh et al. 2021; Qin et al. 2025; Schwarz et al. 2021; Yoshinari et al. 2021). The CRZ neurons also affect feeding indirectly via DILPs by means of colocalized sNPF, indicated by * (Oh et al. 2019). NPF released from EECs of the midgut affects metabolism and aging indirectly via the DILP‐producing cells and JH (Chen et al. 2024) and follicle maturation in ovaries (not shown; Sadanandappa et al. 2021). In adults, there is no prothoracic gland, but in females, ecdysone is produced by the ovaries and regulates female reproduction by promoting biosynthesis of vitellogenin in the fat body (Swevers 2019; Zhu et al. 2021). In males, ecdysone is produced in the male accessory glands (not shown here; Hentze et al. 2013). DILPs regulate production of JH in corpora cardiaca (Tu et al. 2005), and DILPs and JH regulate female reproduction by promoting vitellogenesis in the fat body (LaFever and Drummond‐Barbosa 2005); and both hormones also affect aging (Chen et al. 2024). Note that the insulin‐producing cells that release DILPs receive multiple inputs from other neurons, mediating nutrient status and internal state (see Bisen et al. 2025; Nässel and Vanden Broeck 2016). Hugin neurons innervate the corpora allata, but their function is unknown (Mizuno et al. 2021). This figure is redrawn, from parts of a figure from Kay et al. (2025), with some alterations and updates.

## 8 Discussion

It may not come as a surprise that the functional roles of peptidergic signaling alter and multiply to quite an extent as the developing and growing *Drosophila* larva metamorphoses into an adult fly with its extended repertoire of behaviors. However, this is the first comprehensive comparison of distribution and functional roles of NPHs in these two developmental morphs. As seen in this review, there are of course also many cases where NPHs play similar roles in larvae and adults, and taken together, there is now, for the first time, a catalog of NPH roles over the entire active life cycle of a holometabolous insect (embryonic and pupal functions have been largely ignored here). I have focused on *Drosophila* since numerous studies are available for this species but propose that a similar switch in, and expansion of, functions of NPHs occurs in all holometabolous insects and maybe also in other organisms that undergo complete metamorphosis. Thus, in general, much of the NPH function in larvae involves regulation of growth and development and in the adult, reproduction and social behaviors (Figure 1, Table 1). Some NPH functions are similar in the two stages such as regulation of activity, food seeking, feeding, metabolism, and avoidance behavior, although mechanisms may differ (Table 1). Conserved NPH functions in feeding and metabolism can partly be explained by the fact that development and reproduction both are nutrient‐dependent (see Figure 13). Clearly, it is apparent that the roles of NPHs in larval behavior and physiology, other than those related to growth and development, are understudied and future research is required to enable a more comprehensive comparison to adults.

The *Drosophila* metamorphosis does not affect the total number of different NPHs, except that two NPHs are lost in mature adults, PTTH and EH (Horodyski et al. 1993; McBrayer et al. 2007), and a few bioactive peptides are added in the adult, the male‐specific accessory gland peptides (see Kubli 2003; Wolfner 2002). Not surprisingly, the lost ones are involved in development and the gained ones in reproduction and post‐mating physiology. Instead, the main changes in NPH signaling over metamorphosis are seen in NPH functions and in the neurons/cells producing them. One important post‐metamorphic difference is the appearance of adult‐specific sexual dimorphisms, which are seen also in several neuronal circuits of the CNS and their NPHs (Auer and Benton 2016; Kim and Pan 2026; Rideout et al. 2010). A further important difference resulting from the metamorphosis is the elaboration and rewiring of neuronal circuitry into the highly complex modular circuits of the adult CNS. Especially interneurons are more numerous and elaborate, and in this new circuit context NPH functions are likely to change and diversify.

Functional differences in NPHs in the two stages are easier to assess experimentally for peptide hormones released from NSCs and EECs since these cells can be genetically ablated or functionally manipulated and the effect on physiology and behavior monitored with basic experiments. On the other hand, NPHs in interneurons may contribute to a rich variety of functions depending on the circuit where they are released (Endres et al. 2026), and they commonly act with co‐released fast acting SMNs (classical neurotransmitters; Hökfelt et al. 2018; Merighi 2026; Nässel 2018; Nusbaum et al. 2017; Svensson et al. 2019). Interneuronal neuropeptides are usually modulators of fast synaptic neurotransmission by SMNs and play roles in reconfiguring neuronal circuits in context‐dependent manners (Marder 2012; Nusbaum et al. 2017). The spatial and temporal signaling dynamics of interneuronal NPHs need careful consideration: distance of peptide diffusion, duration of action, network properties of target circuit, and so on (Marder 2012; Merighi 2026; Nässel 2009; Nusbaum and Blitz 2012; Nusbaum et al. 2017; Xia and Li 2025). Since a given interneuronal NPH may act with different outcomes in multiple circuits of the CNS (Endres et al. 2026), a single specific function is unlikely, which makes comparisons between interneuronal neuropeptides in larvae and adults complex. Furthermore, due to the non‐synaptic nature of neuropeptide signaling (volume transmission), the synaptic connectome of the *Drosophila* brain (Dorkenwald et al. 2024) does not provide sufficient information on peptidergic sender‐target neurons, rendering additional analysis necessary. There have so far only been a few attempts to predict peptidergic “wireless connectomes” in *Drosophila* (see, e.g., McKim et al. 2024; Reinhard et al. 2024; Schlegel et al. 2016), and future studies need to address this deficiency with a multipronged approach, before we can fully assess the complexity of peptide function of interneurons and how these change with metamorphosis. In the nematode worm *Caenorhabditis elegans*, the analysis of such a neuropeptidergic “connectome” has advanced much further (Beets and Watteyne 2025; Beets et al. 2023; Ripoll‐Sánchez et al. 2023).

A few brain circuits that employ NPHs display drastic increases in complexity during metamorphosis. One of the most prominent differences is seen in the clock circuits. In *Drosophila* larvae, the only neuropeptide identified in clock neurons is PDF, produced in the four pairs of LNv neurons (Helfrich‐Förster 1997; Wegener et al. 2026). In the other five pairs of larval clock neurons (DN1, DN2, and 5th‐LNv), no NPH has been identified so far (see Wegener et al. 2026). This is in stark contrast to the adult clock circuits where 12 different NPHs have been detected (and further NPHs indicated by transcriptomics) in the approximately 240 clock neurons (Reinhard et al. 2024). Thus, there is a drastic increase in the number and types of peptidergic clock neurons, and in many cases, these neurons possess several colocalized NPHs or even co‐produced SMNs (Kula‐Eversole et al. 2010; Reinhard et al. 2024; Wegener et al. 2026). This is accompanied by an expansion of the clock circuit motifs supporting novel functions (in timing of physiology and behavior) in the more complex daily life of an adult insect (Reinhard et al. 2024; Wegener et al. 2026).

Since the larval clock circuits overlap with those of the visual system, it is also relevant to compare this with the adult visual system, another example of drastically evolved circuitry. Whereas the larval visual system comprises about a dozen interneurons in one hemisphere (Larderet et al. 2017), the adult one consists of approximately 53,000 interneurons of 732 types (Nern et al. 2025). In terms of NPHs, the larval visual system includes only the PDF‐producing LNv, whereas in adults, not less than 14 NPHs have been identified in many different neuron types of the *Drosophila* optic lobe (see Nässel and Zandawala 2019). Unfortunately, peptide function has only been analyzed for PDF neurons in the larval and the adult visual system (see Reinhard et al. 2024; Sprecher et al. 2011; Wegener et al. 2026), and the roles of the other 13 NPHs in adults remain unknown (Nässel and Zandawala 2019). Many of them are likely to be modulators of SMN action in specific synaptic circuits, but some peptidergic neurons originating in central brain centers may modulate context‐dependent activity levels in the visual system. As an example, the widely arborizing SIFamide‐producing neurons that supply processes bilaterally to almost all brain regions, and execute nutritional state‐dependent modulation of feeding, sleep, and reproductive behavior, also innervate the optic lobes (Martelli et al. 2017; Terhzaz et al. 2007). Thus, their global modulatory action may also include vision.

Many of the brain NSCs are examples of how neurons that survive metamorphosis and produce the same NPHs in both stages switch functions. Notable examples are CRZ‐producing LNCs and the IPCs that regulate development and growth in larvae, whereas they regulate physiology and behavior in adults (Tables 5 and 6). However, also many other peptidergic neuron groups make similar switches, like the ones in the VNC that regulate ecdysis motor patterns in larvae but physiology (including diuresis) and behavior in adults (Figure 8, Tables 1 and 4). Further shifts in functions are seen in the roles of sets of neurons and NPHs that regulate JH and ecdysone signaling in larvae and adults to influence feeding, growth, and reproduction (Figure 13; reviewed in Kay et al. 2025). These include AstA, CRZ, Hugin‐PK, ILPs, and NPF, from neurons either in the brain or SEZ or from midgut EECs. The neurons producing these NPHs signal in a nutrient‐dependent manner in both stages, and their actions in different pathways lead to regulation of production of JH in corpora allata in both stages and ecdysone in prothoracic glands of larvae and ovaries of adults. Outcomes of this signaling in larvae affect developmental timing and growth, whereas in adults, it regulates reproduction and reproductive diapause, as well as affects aging and lifespan (Figure 13). Overlapping action by AstA, Hugin‐PK, and NPF regulates feeding in both stages to ensure nutrients required for development and reproduction. Thus, in summary, this set of molecular messengers is released from cells that translate nutritional cues into responses that orchestrate feeding, growth, development, and reproduction (Kay et al. 2025). Cooption of several of the same cell systems for both growth/development and reproduction therefore depends on the involvement of nutrient sensing and feeding in these larval‐ and adult‐specific phenomena.

An example of a minimal change in peptidergic signaling between larvae and adults can be seen in the mushroom body neurons. Although mushroom body circuitry is drastically elaborated in adults, there is in both stages a subpopulation of intrinsic neurons (Kenyon cells) that produce sNPF (Johard et al. 2008; see Figure 14). These neurons are involved in olfactory reward learning in both larvae (Lyutova et al. 2019) and adults (Knapek et al. 2013) via modulation of cholinergic neurotransmission in intrinsic circuits of the mushroom bodies relevant to memory (Barnstedt et al. 2016). Although some earlier studies suggest that certain aspects of larval learning in *Drosophila* are transferred to the adult, a recent study shows that aversive olfactory memory established in the larva does not last through metamorphosis (Poppinga et al. 2026).

**FIGURE 14:**
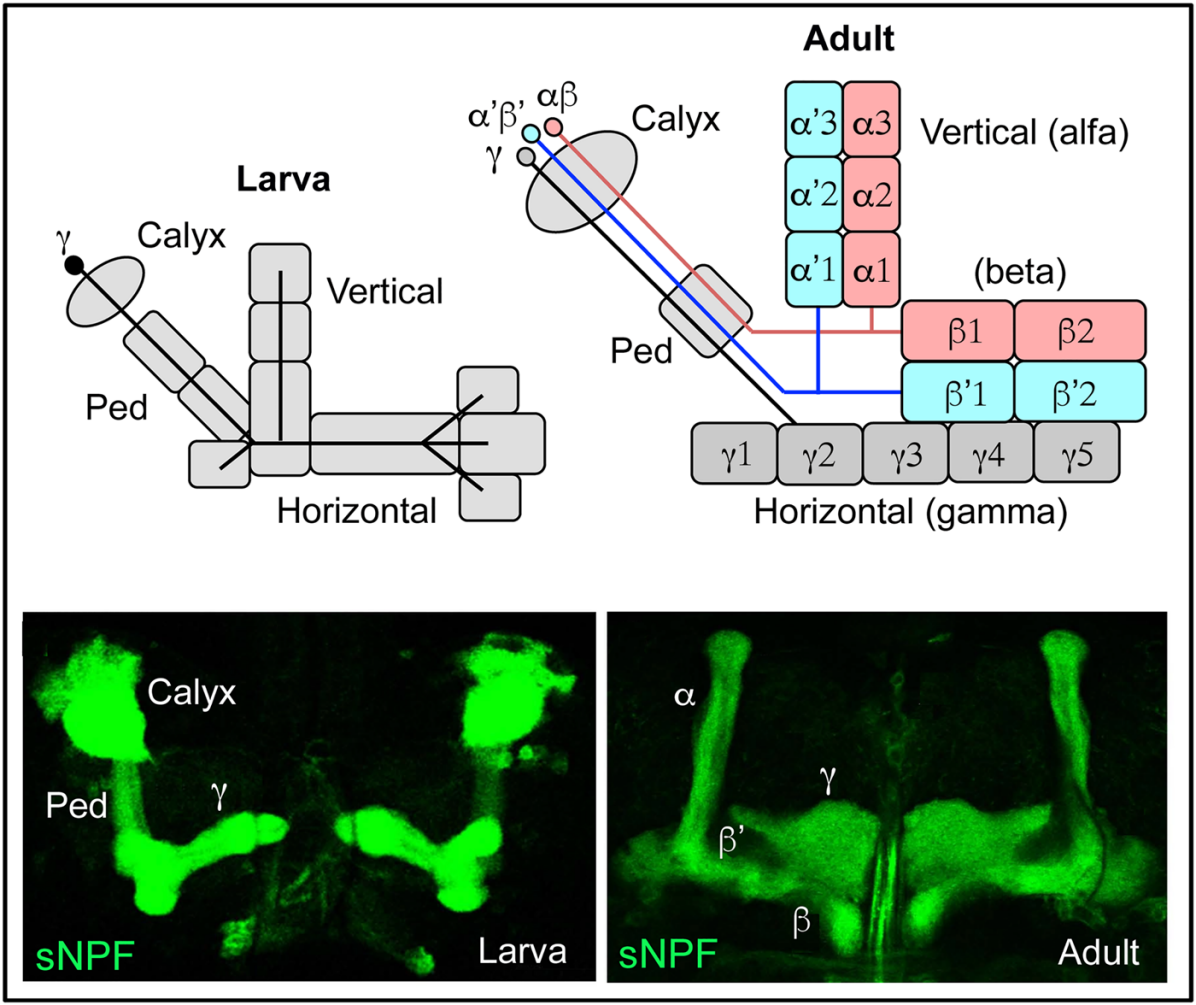
The metamorphosis of the mushroom bodies (MB) and its neuropeptides. The relatively simple larval MB has one major type of intrinsic neurons (γ Kenyon cells) and a few neuronal input and output compartments in the peduncle (Ped), horizontal and vertical lobes (gray boxes). The adult MB has three major types of intrinsic neurons (γ, αβ, and α‘β‘ Kenyon cells) that innervate specific compartments of the lobes (γ, β and γ lobes). This remodeling involves some pruning of larval neurons and addition of novel adult ones. The larval γ neurons regrow and form part of the adult circuitry (the adult γ cells innervate the γ1–γ5 compartments but no longer the vertical lobe). Images are redrawn and simplified from Truman et al. (2023). The bottom panels depict subsets of Kenyon cells producing short neuropeptide F (sNPF), the only known neuropeptide in the MB intrinsic neurons. In larvae, these are the majority of the γ neurons, whereas in adults, sNPF is expressed in subpopulations of γ, β, and γ Kenyon cells but not the α‘β‘ cells. These images are from Nässel et al. (2008) with permission.

Also, the NPHs produced in EECs of the midgut remain the same over metamorphosis, although their cellular distribution pattern in the different regions of the gut differs somewhat (Figure 11). This is remarkable while the midgut undergoes histolysis during metamorphosis and the adult EECs develop *de novo* (see Miguel‐Aliaga et al. 2018). Unfortunately, there is limited information about NPH function of larval EECs; this ranges from regulation of appetite and food seeking, including nutrient sensing (DH31, CCHa2; J. H. Park et al. 2016; Ren et al. 2015), peristalsis of the midgut (DH31; LaJeunesse et al. 2010), immune responses (DH31; Tleiss et al. 2024), and regulation of ecdysone production (Kannangara et al. 2020). In adults, the list is much longer, including nutrient sensing, appetite, food seeking and feeding, metabolism, osmoregulation, stress, sleep, oogenesis, and local gut function (see Tables 3 and 4, with references). Again, larvae are understudied, and one would expect additional functions in larvae, including further roles in development and metabolism. Surprisingly, NPH regulation of diuresis has been ignored altogether in larvae, with one exception: the anti‐diuretic action of ITP on the hindgut (Watanabe et al. 2026).

Finally, several brain centers have received very little attention with respect to NPHs in the two stages. For instance, the central complex and the antennal lobes have been studied to some extent in adults (Carlsson et al. 2010; Epiney et al. 2025; Kahsai and Winther 2011; Sareen et al. 2021; Wolff et al. 2025), but there are no reports on the larval counterparts. Also, the optic lobes of adults remain neglected, except for mapping of some NPHs (see Nässel and Zandawala 2019).

In summary, NPHs are abundant, diverse, and multifunctional messengers that are deployed by a variety of cell types, especially neurons, NSCs, EECs, and other endocrine cells. NPHs display an extensive plasticity in their expression patterns and functions that is both stage‐ and context‐dependent. A given NPH is not only functionally pleiotropic in each developmental morph but also switch and/or expand its repertoire of functions with metamorphosis. In addition, there are substantial taxonomic variations in NPH signaling, even within insects, that further extend this plasticity. Hence, the number of NPH‐encoding genes (and those of corresponding GPCRs) and their expression patterns, as well as functions, differ between insect taxa (see Hauser et al. 2006; Leroy et al. 2026; Nässel and Zandawala 2019; Ragionieri et al. 2021; Schoofs et al. 2017). For the future, it would of interest to determine to what extent the metamorphic changes in NPH signaling described here for *Drosophila* can be extended to other holometabolous insects or even other animals displaying complete metamorphosis. Maybe also in hemimetabolous insects there is a functional change in some of the NHP signaling as the animal enters the final molt and becomes sexually mature and reproductive. Another future project that might be of interest is to summarize the stage‐specific roles of monoamine signaling in *Drosophila*, especially dopamine for which numerous studies are available.

## Data Availability

Data sharing is not applicable to this article as no datasets were generated or analyzed during the current study.
